# Tracing specificity of immune landscape remodeling associated with distinct anticancer treatments

**DOI:** 10.1016/j.isci.2025.112071

**Published:** 2025-02-20

**Authors:** Floriane Cannet, Célia Sequera, Paula Michea Veloso, Abdessamad El Kaoutari, Melissa Methia, Sylvie Richelme, Muge Kaya, Afef Cherni, Mathieu Dupont, Jean-Paul Borg, Christian Morel, Yannick Boursier, Flavio Maina

**Affiliations:** 1 Aix Marseille Univ, CNRS/IN2P3, CPPM, 13009 Marseille, France; 2 Aix Marseille Univ, CNRS, Inserm, Institut Paoli-Calmettes, Centre de Recherche en Cancérologie de Marseille (CRCM), 13009 Marseille, France; 3 Aix Marseille Univ, CNRS, Developmental Biology Institute of Marseille (IBDM), Turing Center for Living Systems, 13009 Marseille, France; 4 Institut Universitaire de France, Paris, France

**Keywords:** Microenvironment, Immune response, Cancer, Machine learning

## Abstract

Immune cells within the tumor microenvironment impact cancer progression, resistance, response to treatments. Despite remarkable outcomes for some cancer patients, immunotherapies remain unsatisfactory for others. Here, we designed an experimental setting using the *Alb-R26*^*Met*^ “inside-out” mouse model, faithfully recapitulating molecular features of liver cancer patients, to explore the effects of distinct anticancer targeted therapies on the tumor immune landscape. Using two treatments in clinical trials for different cancer types, Decitabine and MEK+BCL-XL blockage, we show their capability to trigger tumor regression in *Alb-R26*^*Met*^ mice and to superimpose distinct profiles of immune cell types and immune-checkpoints, impacting immunotherapy response. A machine learning approach processing tumor imaging and immune profile data identified a putative signature predicting tumor treatment response in mice and patients. Outcomes exemplify how the tumor immune microenvironment is differentially reshaped by distinct anticancer agents and highlight the importance of measuring its modulation during treatment to optimize oncotherapy and immunotherapy combinations.

## Highlights

• *Alb-R26*^*Met*^ liver cancer model recapitulates patient molecular and immune profiles
• The distinct action of anticancer drugs on tumor immunity highlights its plasticity
• Distinct anticancer agents differentially reshape the tumor immune landscape
• Distinct treatments partially flatten immune heterogeneity of pre-treated tumors

## Graphical abstract

**Figure:**
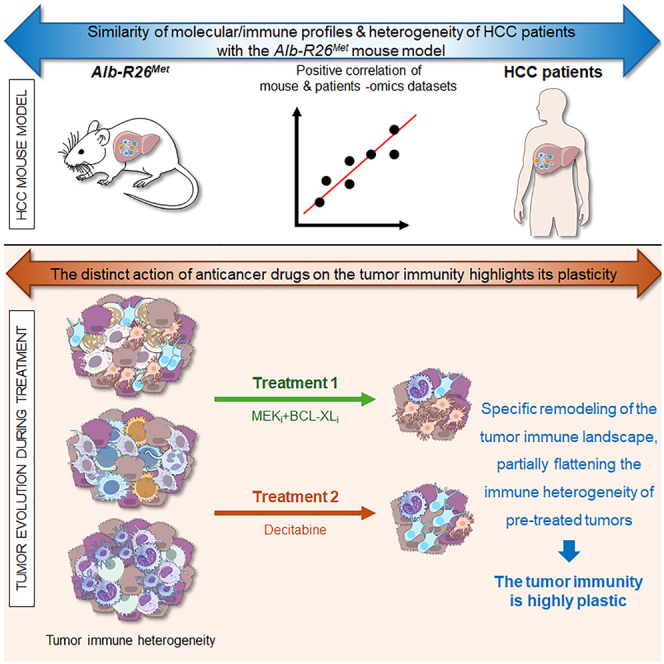

## Introduction

Cancer immunotherapy is rapidly expanding across several cancer types, with encouraging outcomes in subsets of patients. This is paralleled by a growing identification of distinct subtypes of immune cells, each characterized by unique actions on cancer cell recognition and targeting.^1^^,^^2^^,^^3^^,^^4^^,^^5^^,^^6^^,^^7^^,^^8^ The immune composition of the tumor microenvironment, with a permissive state to cytolytic T cell activity, is considered as a key trait to determine the outcomes of an anticancer immune response. Still, cancer cells must be recognized by immune cells, for example, through the expression of cancer-specific antigens, which in most cases arise from genomic alterations. This configuration led to the generation of effective blocking agents to “unmask” cancer cells while potentiating the anticancer action of immune cells. The development of T cell immune-checkpoint inhibitors (ICI) such as antibodies blocking the programmed cell death protein 1 (PD1) or its ligand (PD-L1) and the cytotoxic T-lymphocyte-associated antigen 4 (CTLA-4), has rapidly introduced immunotherapy for clinical management of several cancer types.^9^ Whereas blockage of the PD1/PDL1 system prevents inhibition of T cell function, blockage of CTLA4 induces expansion of tumor reactive T cells.^10^ Additional targeting agents are emerging in relation to the role of other immune modulators involved in cancer cell identification and targeting by immune cell types.^11^^,^^12^^,^^13^^,^^14^ However, the great enthusiasm for immunotherapies is mitigated by clinical trial outcomes illustrating that most patients do not respond to these agents when used as monotherapy whereas responders often develop resistance, besides severe toxicity observed in a subfraction of treated patients. Among reasons responsible for primary or acquired ICI resistance are the presence of immunosuppressive cells, T cell exhaustion, inflammatory phenotypes, and insufficient immunogenic epitopes on cancer cells. Furthermore, the lack of robust genomic and immune signatures to predict responders remains a major concern. Indeed, currently applied signatures, either based on neoantigens, on expression levels of immune-checkpoints, or on the entity of immune subtypes (e.g., CD8^+^:CD4^+^:PD1^+^ T cells), only marginally correlate with response and/or significantly overlap between responders versus resistant patients. A growing number of studies indicate that this steady-state evaluation in naive tumors (before therapy) has strong limitations as it does not consider how tumors with their microenvironment may change during treatment. This is notably relevant in relation to therapies with anticancer agents, which not only trigger cytostatic/cytotoxic effects on cancer cells, but also can drastically impact the expression of new neoantigens by cancer cells, the expression levels of immune-checkpoints, and the immune cell type composition in the tumor microenvironment, leading to immunostimulatory or immunosuppressive states.

We explored how tumors with their microenvironment change in relation to anticancer treatment types in the context of hepatocellular carcinoma (HCC), the most frequent primary liver cancer, the third leading cause of cancer-related mortality.^15^^,^^16^ Despite most HCC can be partially linked to main risk factors, about 10% of cases appear in liver tissue with no identified risks.^17^^,^^18^ HCC arising in the absence of cirrhosis are related to the activation of oncogenes by viral insertions, structural rearrangements, or from the malignant transformation of benign hepatocellular adenomas. Available therapies for clinical HCC management remain largely unsatisfactory. Curative treatments for HCC patients include surgical resection, local ablation, and liver transplantation. Nevertheless, in most patients, HCC is detected at late stages, thus limiting therapeutic options and efficacy. The multi-receptor tyrosine kinase (RTK) inhibitors (RTKi), sorafenib, cabozantinib, lenvatinib, and regorafenib, have been and are still used for HCC treatment, despite the moderate overall survival benefit. Recently, anti-PD1/PDL1 together with anti-VEGFR blockage (atezolizumab+bevacizumab) has been introduced as first-line HCC therapy, and subsequently also anti-PDL1+anti-CTLA4 (durvalumab+tremelimumab).^19^^,^^20^ Nevertheless, treatment benefits are limited to a subset of patients, with the lack of a robust signature for predicting putative responders. Recently, the combinatorial expression of 11 genes has been proposed as a predictive response signature to anti-PD1 therapy for advanced HCC.^21^ Paradoxically, a worsening with exacerbation of tumor growth was observed in about 13% of PD1 patients, a clinical response pattern called hyperprogression.^22^ Of notice, recent clinical trials have reported that combination treatments of ICI with RTKi lead to a higher percentage of overall response rate, with a striking 8% complete response.^23^ These outcomes strengthen the importance of uncovering whether and how anticancer agents, while targeting cancer cells, can impact the tumor immune microenvironment, thus fostering the action of—specific—immunotherapeutic agents.

We have previously illustrated the adequacy of the *Alb-R26*^*Met*^ model to recapitulate several features of liver cancer patients, including the resistance to RTKi used in the clinic, the high heterogeneity of alterations, and the temporal heterogeneity of tumor onset.^24^^,^^25^^,^^26^^,^^27^ Liver tumors in the *Alb-R26*^*Met*^ mice are exclusively HCC.^26^ They develop spontaneously as a result of a progressive deregulation caused by a slightly increase in the expression levels of the wild-type RTK MET in the hepatocytes. The model therefore illustrates an extraordinary capability of subtly increased RTK inputs to render liver cells vulnerable to additional spontaneous alterations, leading to tumor initiation and progression.^26^^,^^27^ The *Alb-R26*^*Met*^ tumors are not addicted to MET (coherent with the subtle increase levels of MET wild-type rather than the (over)expression of a MET oncogenic form) and are characterized by a wide range of molecular alterations (coherent with a broad predisposition to spontaneous alterations at the tumor initiation phases). Through comparative analyses using four independent human HCC cohorts, here we document how the *Alb-R26*^*Met*^ HCC model faithfully recapitulates several molecular features of HCC patients. We additionally illustrate its proficiency to recapitulate as well the heterogeneity of immune cell type composition, with profiles comparable to four HCC patient cohorts.

We reasoned that the *Alb-R26*^*Met*^ “inside-out” mouse genetic setting is a particularly appropriate tool to explore the specificity of distinct anticancer regiments to remodel the tumor immune profile. We document how distinct anticancer agents differentially reshape the tumor immune landscape. Results exemplify its remarkable plasticity in relation to the anticancer treatment used. We quote the consequences of these remodeling configurations on immunotherapy effectiveness. We illustrate how exploring the switch of the tumor immune profile in relation to the anticancer treatment can guide toward optimization of combined therapies. Finally, we propose how a machine learning strategy can provide a signature classifying tumor response to treatment, based on a combinatorial use of three immune cell type parameters.

## Results

### The *Alb-R26*^*Met*^ liver cancer mouse model recapitulates the molecular and immune diversity as well as the complexity of HCC patients

**Figure 1:**
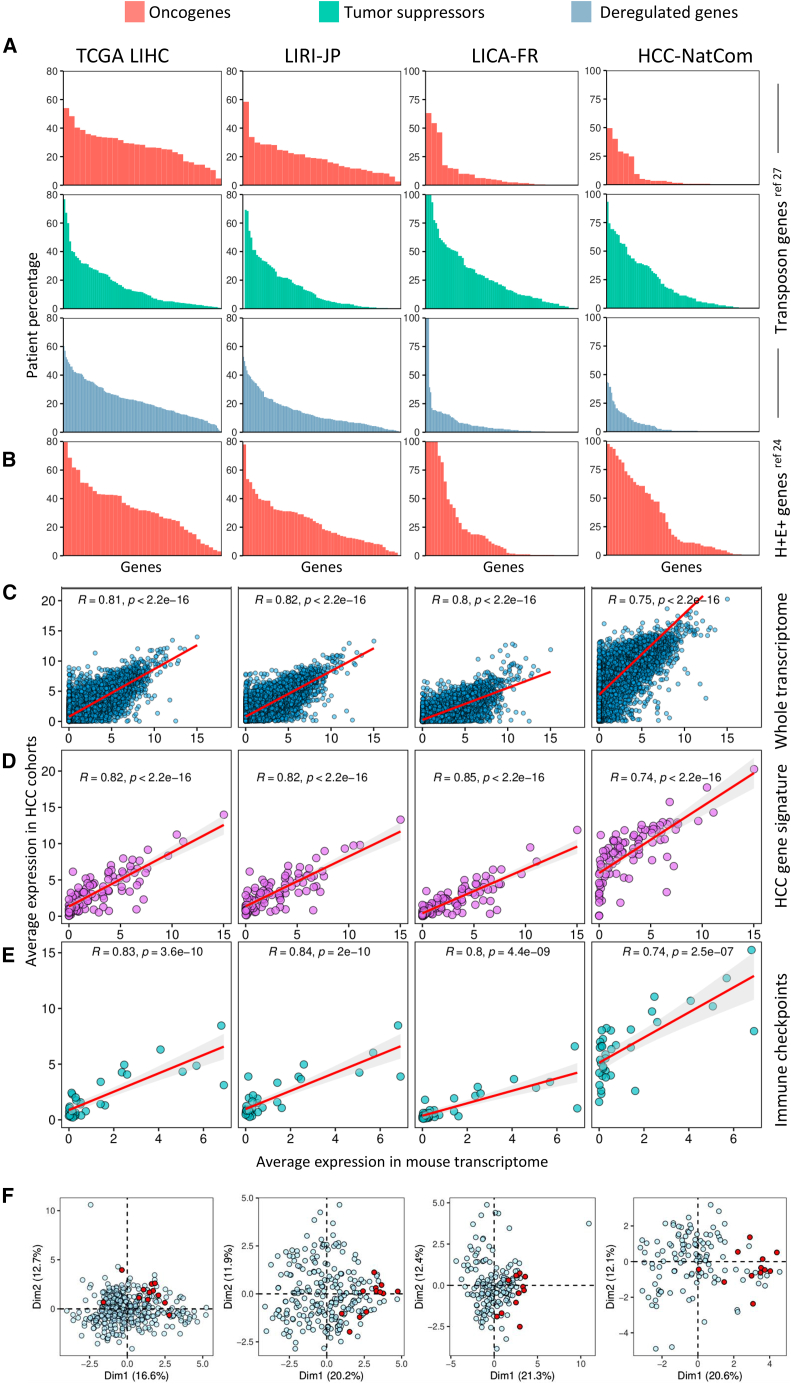
Comparative analysis of deregulated genes, transcriptomic correlations, and immune profile dispersions in the *Alb-R26*^*Met*^ HCC model versus HCC patient cohorts To assess the clinical relevance of the *Alb-R26*^*Met*^ HCC mouse model, -omics outcomes from these mice were compared with transcriptomics data of four independent HCC cohorts (TCGA-LIHC, LIRI-JP, LICA-FR, and HCC-NatCom). Collectively, results illustrate the degree of correlation of *Alb-R26*^*Met*^ with human HCC cohorts, based on which the translatability of findings from the *Alb-R26*^*Met*^ model can be inferred. (A) A transposon mutagenesis screen we previously performed in the *Alb-R26*^*Met*^ genetic setting have identified 29 predicted oncogenes (red), 84 predicted tumor suppressors (green), and 137 deregulated genes (blue).^27^ The bar plots illustrate the percentage of patients with overexpression of predicted oncogenes, downregulation of predicted tumor suppressors, and altered expression of deregulated genes, for each cohort. (B) Through methylome studies we previously performed in the *Alb-R26*^*Met*^ genetic setting, we have identified 55 genes simultaneously hypermethylated in gene body and overexpressed (H+E+), qualified as oncogenes (red).^24^ The bar plots illustrate the percentage of patients with overexpression of these oncogenes in each patient cohort. (C–E) Correlation analyses between the average gene expression in the *Alb-R26*^*Met*^ mouse model and in four independent HCC patient cohorts. Scatterplots depict the relationship between the average expression of genes in the mouse transcriptome and patient samples. Each panel displays the *Pearson* correlation coefficient R and the associated *p* value to quantify the strength of the relationship. The regression line (red) represents the best fit, with a shaded region indicating the 95% confidence interval. The consistently high R values across all comparisons underscore the agreement between mouse and human datasets. (C; blue): correlation of the whole transcriptome across all genes. (D; purple): correlation based on a 96 HCC gene signature. (E; teal): correlation of immune-checkpoint expression levels. (F) PCA biplots showing the dispersion of the *Alb-R26*^*Met*^ mouse immune profile (red dots) among patient samples in each cohort. The input data consist of a deconvolution matrix predicting immune enrichment scores. The axes (Dim1 and Dim2) represent the principal components explaining the variation in immune enrichment profiles, with the mouse immune profile clustering among patient samples.

A feature conferring uniqueness to the *Alb-R26*^*Met*^ “inside-out” cancer model is its ability to recapitulate, in an immune competent context, the temporal heterogeneity of tumor onset and the molecular heterogeneity of human HCC patients.^24^^,^^26^^,^^27^ All *Alb-R26*^*Met*^ tumors are exclusively HCC (Figure S1; Table S1), as previously reported.^26^ Notably, tumors have distinct morphological/histological/immune characteristics, even when present in the same liver lobe of a given mouse, thus qualifying them as distinct tumors (Figure S1; Table S1). To assess the clinical relevance of the *Alb-R26*^*Met*^ model, we compared its -omics outcomes with four independent HCC patient cohorts (TCGA, LICA, LIRI, HCC-NatCom). Through a transposon mutagenesis screening we previously performed in the *Alb-R26*^*Met*^ genetic setting, we identified 29 predicted oncogenes, 84 predicted tumor suppressors, and 137 deregulated genes.^27^ Figure 1A reports, for each cohort, the percentage of HCC patients in which these genes are altered (percentage of genes altered in at least 10% of patients: 77% in TCGA LIHC; 56% in LIRI-JP; 39% in LICA-FR; 35% in HCC-NatCom; Data S1). Through methylome and transcriptome analyses we previously performed in *Alb-R26*^*Met*^ tumors, we identified 55 genes overexpressed and hypermethylated in the gene body.^24^ Figure 1B reports, for each cohort, the percentage of HCC patients in which these genes are upregulated (percentage of genes altered in at least 10% of patients: 87% in TCGA LIHC; 76% in LIRI-JP; 43% in LICA-FR; 60% in HCC-NatCom; Table S2).

In the present study, we performed a bulk RNA-seq analysis of *Alb-R26*^*Met*^ tumors and control livers (Table S3, Data S2). Principal-component analysis (PCA) reports a separate clustering of tumors versus control livers (Figure S2A). Of note, *Alb-R26*^*Met*^ tumors showed inter-variability, exemplifying HCC heterogeneity. Next, we performed correlation analyses comparing the transcriptome of HCC *Alb-R26*^*Met*^ mice and patients. Figure 1C shows strong positive correlations in the four cohorts, with *Pearson*’*s* coefficients *R* ranging from 0.75 to 0.82. We found similar correlations when analyzing 96 markers characterizing HCC,^26^ with coefficients ranging from 0.74 to 0.84 (Figure 1D; Table S4). Moreover, focusing on the expression of 36 immune-checkpoints (both co-inhibitory and co-stimulatory signals), we obtained again strong positive correlations between mouse and human HCC, with coefficients ranging from 0.74 to 0.84 (Figure 1E; Table S5). The immune profile of the *Alb-R26*^*Met*^ tumors was further compared with that of human HCC patients by performing deconvolution of bulk RNA-seq data. Figure 1F reports, for each cohort, the PCA plots of HCC patients (in blue) and of *Alb-R26*^*Met*^ HCC (in red), documenting the overlap of analyzed *Alb-R26*^*Met*^ HCC with a proportion of HCC patients.

**Figure 2:**
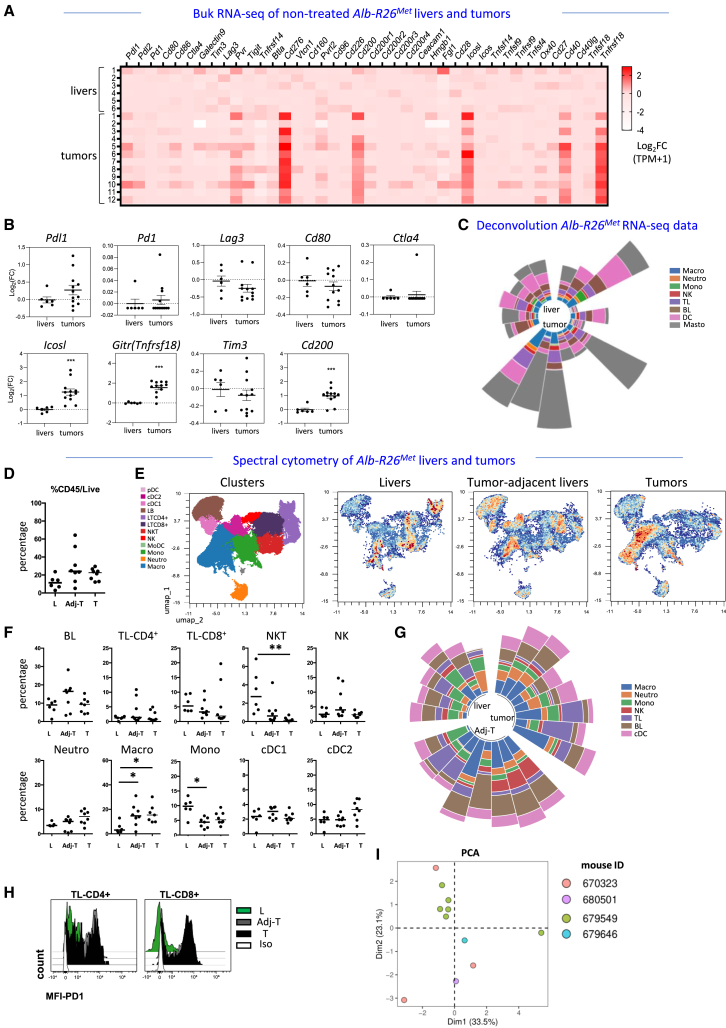
The tumor immune cell type composition in the *Alb-R26*^*Met*^ liver cancer model recapitulates patient heterogeneity (A and B) Heatmap (A) and dotplots (B) reporting the expression levels of the indicated immune checkpoints (log_2_FC) in *Alb-R26*^*Met*^ non-tumoral livers (*n* = 6) and advanced HCC tumors (*n* = 12). (C) Circular plot illustrating the heterogeneity of immune cells population based on deconvolution of RNA-seq data of *Alb-R26*^*Met*^ non-tumoral livers and tumors. (D–I) Spectral cytometry analysis of liver from non-tumor bearing mice (L; *n* = 6), tumor-adjacent tissue (Adj-T; *n* = 8), and tumors (T; *n* = 8) from *Alb-R26*^*Met*^ mice. (D) Percentage of CD45^+^ cells among total live cells. (E) UMAP showing unsupervised analysis of CD45^+^ cells from *Alb-R26*^*Met*^ livers, tumor-adjacent livers, and tumors. Overlay of all samples showing immune cell clusters with the corresponding color-code (left) density map of each indicated tissues (right). Note the different “switch on” patterns in the three microenvironments. (F) Quantifications of the indicated immune cell population among CD45^+^ cells. (G) Circular plot illustrating the heterogeneity of immune cells population. (H) Histograms showing the Median Fluorescent Intensity (MFI) of PD1 at the surface of CD4^+^ and CD8^+^ TLs in livers from non-tumor bearing mice (L; green), tumor-adjacent livers (Adj-T; gray), and tumor (T; black). Isotype control (Iso) is shown in white. (I) PCA of the abundance of immune cell population, based on spectral cytometry data, of different non-treated tumors from the indicated *Alb-R26*^*Met*^ mice (identical colors indicate tumors from the same mouse). Statistical analyses were performed using Kruskal-Wallis. Levels of significance: ∗*p* ≤ 0.05; ∗∗*p* ≤ 0.01; ∗∗∗*p* ≤ 0.001. Bars represent SEM and notches median levels.

We further extended these analyses to determine whether the *Alb-R26*^*Met*^ cancer model recapitulates the heterogeneity observed in human HCC patients. Concerning immune-checkpoints, we found rather heterogeneous expression levels across different *Alb-R26*^*Met*^ tumors, including *Pdl1*, *Pd1*, *Lag3*, *Pdl2*, *Cd80*, *Tnfrsf18* (*Gitr*), *Tim3*, *Icosl*, *Tigit*, *Cd40*, *Cd200*, *Cd276*, *Pvr*, *Cd86*, *Ox40L (Tnfsf4)*, *Galectin9* (Figures 2A–2B, S2B, and Data S3). Concerning immune cell types based on bulk RNA-seq deconvolution, we found as well heterogeneous patterns characterizing distinct *Alb-R26*^*Met*^ tumors (Figures 2C, S2C; the pattern of distinct cell types in the analyzed samples are reported in Figure S2D). The immune cell type composition in the *Alb-R26*^*Met*^ liver tumors was additionally explored at anatomo-histological levels on sections, quantifying and localizing resident macrophages, infiltrated lymphocytes, and neutrophils. Outcomes revealed again heterogeneous patterns of immune cell infiltrations between control livers and tumors (Table S1).

To further extend the characterization of the immune cell type composition in the *Alb-R26*^*Met*^ tumors, mice were sacrificed and dissected tumors were processed to obtain single cell suspension for spectral cytometry analysis with a designed antibody panel (Table S6; an example of supervised gating strategy to identify immune cells is shown in Figure S3A). Combining supervised and unsupervised analyses projected through uniform manifold approximation and projection (UMAP) clustering (see Method section), we could identify and quantify T lymphocytes (TLs) CD4^+^ and CD8^+^, B lymphocytes (BL), Natural Killer (NKs), NK lymphocytes (NKT), neutrophils, macrophages, monocytes, classical dendritic cells (cDCs) type 1 (cDC1) and type 2 (cDC2), monocyte-derived DCs (MoDC), and plasmacytoid DCs (pDCs; Figures 2D–2G, S3B and S3C). Despite we did not find significant differences in the infiltration of CD45^+^ leukocytes (Figure 2D), we found a significant increase of macrophages and decrease of NKT in *Alb-R26*^*Met*^ tumors compared with control livers from non-tumor-bearing mice (Figures 2E–2F, S3B and S3C). Results again illustrate a heterogeneity in the immune cell type composition across different *Alb-R26*^*Met*^ tumors (Figure 2G). We additionally analyzed the expression of the immune-checkpoint PD1 at the surface of TL-CD4^+^ and TL-CD8^+^. We found a high expression of PD1 in *Alb-R26*^*Met*^ tumors and liver-adjacent tumors compared to liver controls (Figure 2H). Finally, using the abundance of immune cell infiltration among CD45^+^ immune cells, we analyzed by PCA the clustering of different *Alb-R26*^*Met*^ tumors dissected from the same mouse (each mouse is indicated with a color code). Results shows tumor heterogeneity in the immune composition both from different mice and among those found in the same mouse (Figure 2I). Together, these findings demonstrate that, despite limitations that all genetic mice can have to fully match a human pathology, the *Alb-R26*^*Met*^ HCC model recapitulates several molecular and immune features of HCC patients, representing the diversity and complexity of human HCC. Results also illustrate a remarkable heterogeneity on the immune cell types and on the expression levels of immune-checkpoints in the *Alb-R26*^*Met*^ tumors, which is another characteristic of human HCC patients. This comprehensive characterization qualifies the *Alb-R26*^*Met*^ mice as a relevant cancer model for studying immune cells in spontaneous tumors during their progression and for investigating how anticancer treatments may reshape the immune landscape.

### Both Decitabine and MEK+BCL-XL inhibition trigger tumor regression in the *Alb-R26*^*Met*^ liver cancer model

The effectiveness of currently available HCC therapies is largely unsatisfactory, with a percentage of responding patients below 20%,^28^ underlying the need to develop new therapeutic options. The possibility to repurpose promising treatments currently explored in clinical trials for other complex cancers, uncovering their effects on the tumor immune profile to identify optimal immunotherapies to be associated, is among strategies that deserves to be explored. Using the *Alb-R26*^*Met*^ cancer model, resistant to several RTKi used in the clinic,^26^^,^^29^ we previously reported two distinct types of molecular targeted therapies. One is based on an epigenetic modulator, the de-methylating agent Decitabine, used in the clinics for other cancer types and in 470 clinical trials (https://clinicaltrials.gov/search?cond=Cancer&term=Decitabine). We and others have previously showed that in HCC cells and mouse preclinical models, Decitabine reduces tumorigenicity by decreasing levels of oncogenic signals,^24^^,^^30^^,^^31^ including the oncogene ADAMTSL5.^29^ Additionally, the Decitabine target DNMT3 has been reported to be among the four-driver gene signature for HCC prediction.^32^ The other treatment is based on combined MEK and BCL-XL blockage (MEK_i_+BCL-XL_i_), a synthetic lethal interaction we uncovered on a panel of human/mouse HCC cells and demonstrated to be effective *in vivo*,^26^^,^^33^ which has been reported also in other cancer types.^34^^,^^35^^,^^36^^,^^37^ We showed that a phosphoERK/BCL-XL/MCL1 signature, deregulated in *Alb-R26*^*Met*^ tumors, characterizes a subgroup of HCC patients with poor prognosis.^26^ The therapeutic strategy based on a combinatorial blockage of MEK+BCL-XL/BCL2 is an active ongoing clinical trial for treating patients with advanced or metastatic solid tumors (https://clinicaltrials.gov/study/NCT02079740?cond=Solid%20Tumors&term=bcl-XL&limit=25&rank=3) and with recurrent ovarian and endometrial cancers (https://clinicaltrials.gov/study/NCT05691504?cond=Cancer&term=pelcitoclax&rank=2). We therefore explored whether and how Decitabine and MEK_i_+BCL-XL_i_ impact the tumor immune microenvironment, thus influencing the choice of optimal immunotherapy for combinatorial treatments.

**Figure 3:**
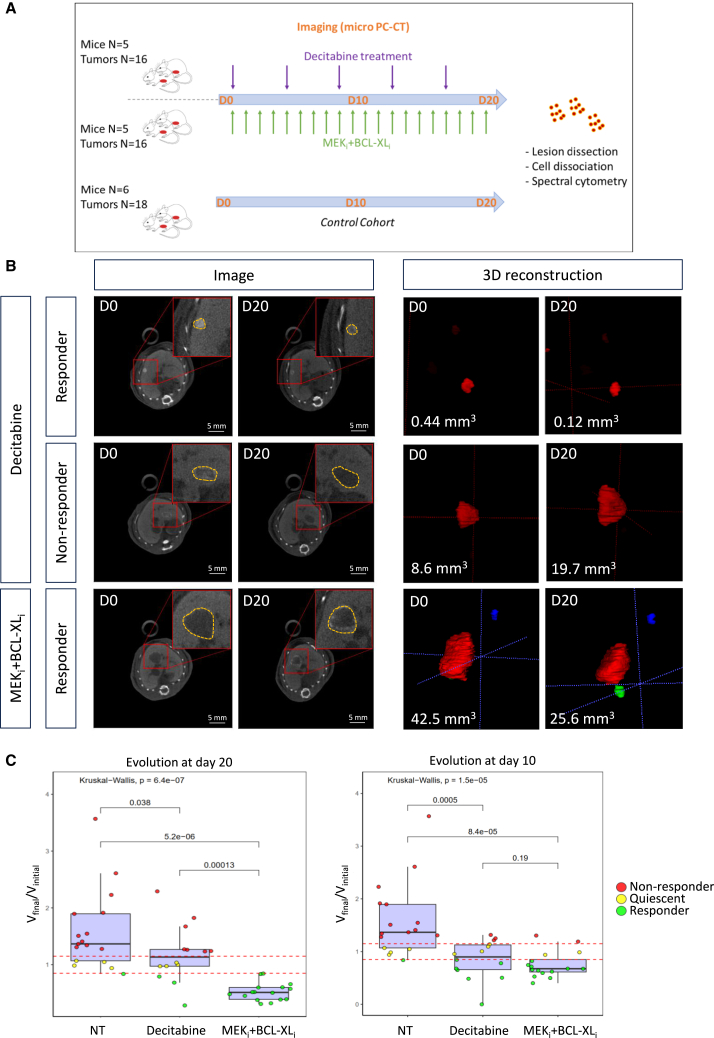
Longitudinal *in vivo* imaging to assess the effects of Decitabine and MEK+BCL-XL blockage on endogenous tumors in the *Alb-R26*^*Met*^ liver cancer model (A) Schematic representation of the protocol we followed to assess the treatment effects of Decitabine and MEK+BCL-XL blockage in the *Alb-R26*^*Met*^ liver cancer model, compared to untreated mice. Three cohorts were used: untreated as control (mice *n* = 6), treated with Decitabine (every 4 days; mice *n* = 5) and the other treated with MEK_i_+BCL-XL_i_ (every day; mice *n* = 5). The total number of analysed tumors in each cohort is reported. (B) Left: examples of PC-CT imaged tumors, before and after the indicated treatments. Right: 3D reconstruction of the tumor after the segmentation. Examples of responding and non-responding tumors are illustrated for Decitabine cohort, whereas only a responder example is shown for MEK_i_+BCL-XL_i_ as all tumors responded to treatment. (C) Dot-plots illustrating the evolution of the tumor volume after 20 (D20; left) and 10 (D10; right) days for non-treated and treated tumors. Tumors are considered responding if the ratio is less than 0.85, non-responding if it is greater than 1.15, and quiescent between these two thresholds. Dots for the untreated cohorts are reported in both graphs for comparison purposes. Each boxplot shows the distribution of data at 25^th^ percentile, 50^th^ percentile (median), 75^th^ percentile, minimum and maximum values. Dots outside are potential outliers. Statistical analyses were performed using one sided t test and Kruskal-Wallis. Levels of significance is indicated in the panels.

The overall experimental procedure we designed is shown in Figure 3A. Briefly, *Alb-R26*^*Met*^ mice were imaged by photon-counting micro-computed tomography (PC-CT)^33^ to identify those carrying tumors. Tumor-bearing mice were subdivided into three cohorts, one untreated used as control, and two others receiving for a period of 20 days (D20) either Decitabine or MEK+BCL-XL inhibitors (MEK inhibitor: trametinib; BCL-XL inhibitor: ABT-737; Tables S7 and S8). A longitudinal follow-up of tumor evolution over time was performed by PC-CT at D10 and D20 (Figure 3A). Longitudinal PC-CT imaging and data processing through a deep learning model we developed for 3D reconstruction of tumors allowed us to quantify the volume kinetic of treated tumors and the treatment response expressed as the (final volume)/(initial volume) ratio (V_final_/V_initial_). Inspiring us from the mRECIST criteria used in the clinic for HCC patient under treatment, which takes into account the response of all nodules as a whole, we applied a more stringent evaluation analyzing the behavior of each tumor independently (Figure S4A). This choice is also in relation to the fact that each tumor has a distinct profile and behavior, even when in the same liver lobe (Table S8). According to these parameters, we segregated tumors as either responders, non-responders, or quiescent (Figure 3B). We found no correlation between initial volume and regression in either of the treatments (Spearman coefficient: 0.19; *Pearson* coefficient: 0.015), indicating that the response of tumors to the tested treatments is linked to their intrinsic molecular and/or cellular features rather than to their initial size (Figure S4B). Furthermore, we found that combined MEK+BCL-XL blockage elicits a high rate of response (16/16 responding tumors at the end of the treatment), whereas Decitabine leads to a more heterogeneous response (4/16 responding tumors, 4/16 quiescent, and 8/16 non-responding tumors at the end of the treatment (D20); Figure 3C, left). By comparing the tumor response (V_final_/V_initial_) at D10 in *Alb-R26*^*Met*^ treated versus non-treated (NT) mice, we observed that either treatment with Decitabine or MEK+BCL-XL inhibitors already stopped tumor growth or led to their regression as compared with NT tumors (Figure 3C, right). Nevertheless, the number of regressing tumors in both treatment groups further increase at D20 (Figure 3C, left). Notably, MEK+BCL-XL blockage leads to a more pronounced reduction of the tumor volume compared to Decitabine treatment, and all tumors regress (Figure 3C, left). These outcomes show that both treatment types elicit tumor response in the *Alb-R26*^*Met*^ cancer model, with MEK_i_+BCL-XL_i_ particularly effective. Furthermore, results indicate that MEK+BCL-XL blockage triggers a response overtaking the intrinsic tumor heterogeneity on molecular alterations and immune profiles.

### Different treatments superimpose a specific type of immune remodeling on an initial immune profile in the *Alb-R26*^*Met*^ tumor model

Despite efforts to identify putative molecular signatures correlating with ICI response in advanced HCC,^21^^,^^38^ results remain largely unsatisfactory. Emerging evidence in other cancer types has shown that radio/chemo-therapies can drastically change the tumor immune profile observed before treatments.^39^^,^^40^ We therefore explored whether and to what extent distinct molecular targeted therapies can selectively modify the tumor immune profiling in the context of HCC. This knowledge could inspire the design of relevant onco- and immuno- combinatorial therapies. The *Alb-R26*^*Met*^ cancer model is particularly pertinent to address this question for its unicity, notably in relation to the heterogeneity of the immune profile across distinct tumors (Figure 2). Two hypotheses could be made: (1) the initial immune profile (in untreated tumors) is robust and therefore not influenced by the type of anticancer treatments; (2) distinct treatments superimpose a predominant type of remodeling, surpassing the initial immune heterogeneity of untreated tumors. We experimentally explored these two hypotheses by comparing the immune cell profile of *Alb-R26*^*Met*^ tumors either untreated or treated with Decitabine versus MEK+BCL-XL blockage.

**Figure 4:**
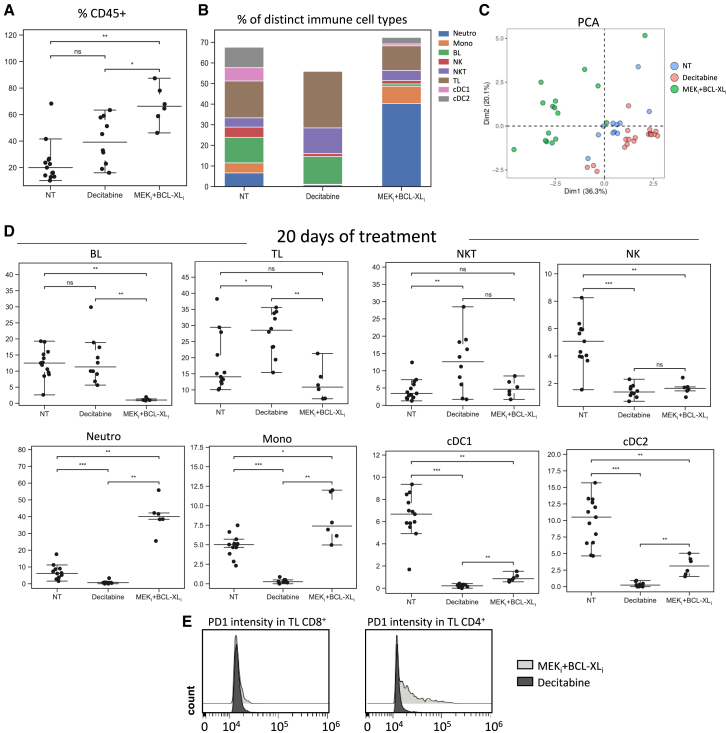
Spectral flow cytometry analysis revealed distinct immune-microenvironment remodeling triggered by Decitabine versus MEK+BCL-XL blockage in the *Alb-R26*^*Met*^ tumors (A) Dot-plots reporting the percentage of CD45^+^ over total number of live cells in untreated (NT), Decitabine and MEK_i_+BCL-XL_i_ treated tumors. (B) Graph reporting the distribution of distinct immune cell types in the indicated groups. (C) PCA of the abundance of immune cell population, based on spectral cytometry data, of non-treated or treated *Alb-R26*^*Met*^ tumors. (D) Dot-plots reporting immune cell types in non-treated, Decitabine or MEK_i_+BCL-XL_i_ treated tumors analyzed at D20. Note the drastic and specific change in the immune cell composition linked to each treatment type. Graphs report on the Y axis the percentage of distinct immune cell types indicated on the top of each graph. (E) Histograms showing the expression of PD1 at the surface of tumor-infiltrating TL CD8^+^ and TL CD4^+^ after 20 days of Decitabine (dark gray) or MEK_i_+BCL-XL_i_ (light gray) treatments. Statistical analyses were performed using Kruskal-Wallis. Levels of significance: ∗*p* ≤ 0.05; ∗∗*p* ≤ 0.01; ∗∗∗*p* ≤ 0.001. Bars represent SEM and notches median levels.

At D20 after imaging, treated mice were sacrificed and dissected tumors were processed for spectral cytometry analysis with an antibody panel (Table S6). Quantification of the percentage of different CD45^+^ cells revealed an increase of the immune infiltration in treated compared to untreated tumors. This increase was significantly higher in MEK_i_+BCL-XL_i_ compared to untreated or Decitabine (Figure 4A). We also observed that, despite some intra-group heterogeneity in the composition of the immune infiltrate (Figure S4C), different treatments induced distinct immune cell remodeling (Figure 4B). Notably, PCA analysis revealed that tumors treated with MEK+BCL-XL blockage clearly separate from untreated and Decitabine group (Figure 4C). Specifically, in MEK_i_+BCL-XL_i_ treated tumors, we found a marked increase of neutrophils (40% of CD45^+^ cells) and monocytes compared with Decitabine treatment or untreated controls (Figures 4B and 4D). We additionally found a significant increase of BLs and TLs together with a higher proportion of classical cDC1 and cDC2 in MEK_i_+BCL-XL_i_ compared with Decitabine treated tumors (Figure 4D). Furthermore, we could observe PD1 expression only in TL CD4^+^ and not in TL CD8^+^, when treated with MEK_i_+BCL-XL_i_ (Figure 4E), possibly indicating an efficient resolution of the disease, supported by the sizable overall regression of these tumors. In summary, after MEK+BCL-XL blockage, tumors have a marked myeloid infiltration whereas Decitabine is characterized by an increase in lymphocyte recruitment. In order to determine the impact of drug treatment in livers, we additionally explored the effects of Decitabine and MEK_i_+BCL-XL_i_ on the immune cell type composition in the liver of tumor-free mice (Figure S5A). Results show changes consistent with those we found in tumors (Figures S5B–S5D). We found a significant decrease of BL, TL (CD8^+^), and NKT alongside a notable increase of neutrophils, monocytes, MoDC, and cDC1 in MEK_i_+BCL-XL_i_ compared with Decitabine treated-mice (Figure S5). Collectively, these findings illustrate how distinct treatments, exemplified here by Decitabine versus MEK+BCL-XL blockage in the *Alb-R26*^*Met*^ cancer model, can drastically modify the immune cell profile in the tumor microenvironment (and in the non-tumoral liver tissue). Furthermore, despite the heterogeneity on the immune cell type composition across different tumors (Figure 4), results indicate that a given treatment partially overcome this immune heterogeneity, imposing a profound remodeling for certain immune cell types. This is exemplified here by the effects of both treatment on cDC1/2, NKs, of Decitabine on monocytes, neutrophils, and of MEK+BCL-XL blockage on BLs (Figure 4D).

### Effects of PDL1+CTLA4 blockage on tumor response in combination with either Decitabine or MEK_i_+BCL-XL_i_

**Figure 5:**
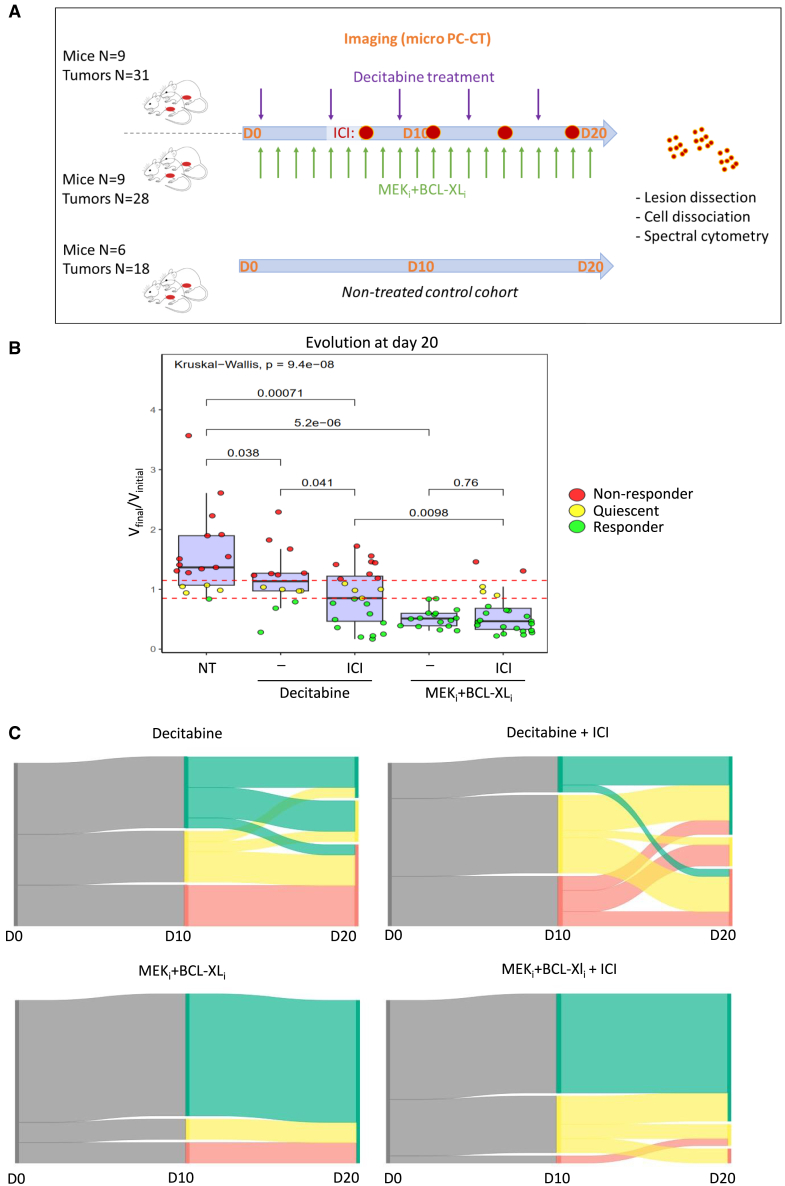
Effects of PDL1+CTLA4 blockage in the context of Decitabine or MEK+BCL-XL inhibition in the *Alb-R26*^*Met*^ liver cancer model (A) Schematic representation of the protocol we followed to assess the effects of anti-PDL1 plus anti-CTLA4 combined with either Decitabine (*n* = 9 mice) or MEK+BCL-XL blockage (*n* = 9 mice) versus untreated controls (*n* = 6 mice) in the *Alb-R26*^*Met*^ liver cancer model. After 7 days of Decitabine or MEK+BCL-XL treatment, anti-PDL1 plus anti-CTLA4 were co-administered every four days (indicated in the scheme with red dots). Mice were imaged with PC-CT at D10 and D20 to follow tumor evolution and to quantify tumor volume. At D20, tumors were dissected and dissociated cells were processed to analyze the immune cell type population by spectral flow cytometry. The total number of analysed tumors in each cohort is reported. (B) Dot-plot reporting the evolution of the tumor volume at D20. Dots for untreated, single Decitabine, or BCL-XL+MEK cohorts, already shown in Figure 3C, are reported here for comparison purposes. Each boxplot shows the distribution of data at 25^th^ percentile, 50^th^ percentile (median), 75^th^ percentile, minimum and maximum values. Dots outside are potential outliers. Statistical analyses were performed using one sided t test and Kruskal-Wallis. Levels of significance are indicated in panels. (C) Sankey diagrams illustrating the overall evolution of tumors classified according to their behavior as regressive (green), quiescent (yellow), and evolutive (red) at D10 and D20. Each diagram corresponds to the four treated cohorts (Decitabine or MEK_i_+BCL-XL_i_, with or without immunotherapies).

The distinct immune cell type remodeling triggered by different anticancer treatments, exemplified here by Decitabine versus MEK_i_+BCL-XL_i_, prompted us to investigate its impact on the action of ICI agents. To address this question, we selected the type of ICIs based on the following considerations: (1) anti-PD1/PDL1 is currently used as first-line treatment in HCC^19^^,^^20^; (2) anti-PDL1 plus anti-CTLA4 has been recently added to the BCLC guidelines as first line of HCC treatment^19^^,^^41^; (3) PD1/PDL1 and CTLA4 with the corresponding CD80 and CD86 receptors are expressed in *Alb-R26*^*Met*^ tumors with heterogeneous levels (Figures 2B and S2B). For this purpose, a new group of *Alb-R26*^*Met*^ tumor-bearing mice was generated to constitute cohorts treated with a combinatorial regiment of anti-PDL1+anti-CTLA4 together with either Decitabine or MEK+BCL-XL blockage (Figure 5A; Tables S7 and S8). Intriguingly, we found that PDL1+CTLA4 blockage elicits different effects depending on the anticancer treatment tested (Figure 5B). The overall evolution of tumors from D0 to D20 in each cohort is illustrated by Sankey diagrams (Figure 5C). We observed a significant increased number of responding tumors when PDL1+CTLA4 blockage is combined with Decitabine versus Decitabine alone (Figures 5B and 5C). Instead, we observed no significant effects of PDL1+CTLA4 blockage when combined with MEK_i_+BCL-XL_i_, rather with the emergence of some non-responding tumors (Figures 5B and 5C).

**Figure 6:**
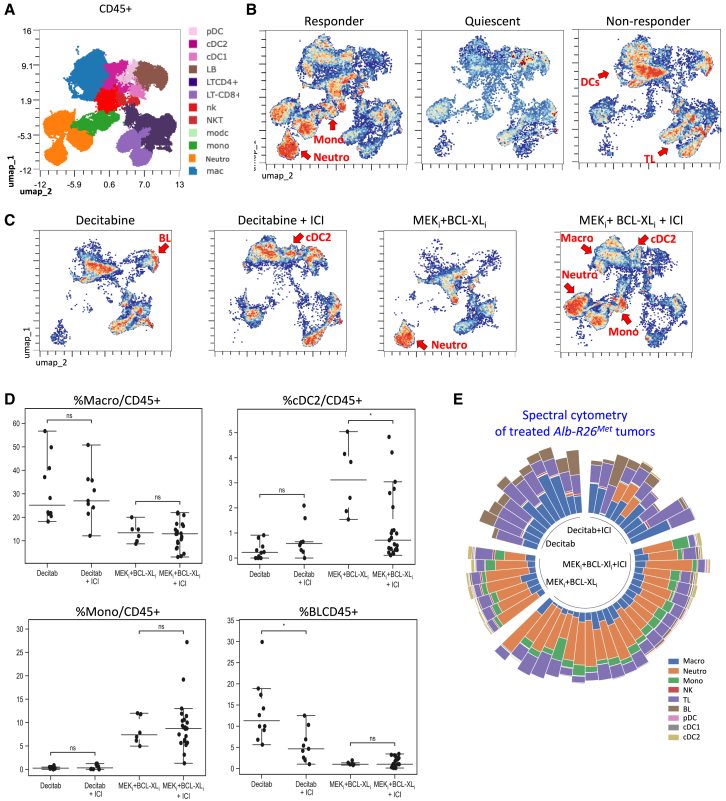
Spectral cytometry analysis revealed distinct immune cell type clusters in relation to tumor behavior and treatment types (A) Cluster identification results from marker expression levels in tumors analyzed by spectral flow cytometry at D20. The different immune cell clusters are reported on the right. (B) UMAP clustering corresponding to responding, quiescent, and non-responding tumors. Note the different “switch on” patterns of responder and non-responder tumors versus the “switch off” pattern of quiescent tumors. Responding tumors are characterized by a “switched on” pattern of neutrophils and monocytes, whereas non-responders by a “switched on” pattern of cDC2s and TLs. (C) UMAP clustering of each cohort corresponding to cells from tumors dissected from *Alb-R26*^*Met*^ mice treated with Decitabine or MEK_i_+BCL-XL_i_, without or with PDL1+CTLA4 blockage. (D) Dot-plots reporting the percentage of the indicated immune cell types in tumors from mice exposed to the indicated treatments. (E) Circular plots illustrating the heterogeneity of immune cells after treatment at D20 in the *Alb-R26*^*Met*^ mouse model, grouped according to the treatment type. Statistical analyses were performed using Kruskal-Wallis. Levels of significance: ∗*p* ≤ 0.05; ∗∗*p* ≤ 0.01; ∗∗∗*p* ≤ 0.001. Bars represent SEM and notches median levels.

At D20, tumors were dissected and dissociated cells were analyzed by spectral cytometry. UMAP clustering revealed a distinct composition of the immune cell type populations within the tumor microenvironment, in relation to tumor behavior and to the type of treatment (Figures 6A–6D). Remarkably, we found a distinct on/off pattern of immune cell clusters characterizing tumors in relation to their response behavior, irrespectively on the treatment type (Figure 6B). Responding tumors showed a “switched on” pattern of neutrophils and monocytes in contrast to non-responding tumors with a “switched on” pattern of clusters corresponding to cDC2s and TLs (Figure 6B). Although it is generally considered that neutrophil abundance in tumors correlate with poor prognosis, it has been recently reported that an acute expansion of neutrophil numbers with a distinct state correlates with effective therapy.^42^ This is further supported by other studies illustrating that activated T cells can recruit neutrophils to kill cancer cells,^43^ and that sorafenib-responding patients had a higher percentage of neutrophils.^44^ Instead, tumors classified as quiescent do not show any hot regions in the UMAP clustering used for dimension reduction. Thus, in quiescent tumor, cancer cells might not be visible to the immune system. Furthermore, we found that combining Decitabine with ICI treatment decreases the BL cluster compared to Decitabine alone (Figures 6C–6D, S6A and S6B). Instead, combining MEK+BCL-XL inhibition plus ICI treatment enriches the tumor microenvironment with neutrophil, monocyte, and macrophage clusters (Figures 6C–6D, S6A and S6B). Intriguingly, when analyzing the samples individually, we observed that the addition of ICI treatment leads to a higher heterogeneity among samples, irrespectively when combined with Decitabine or MEK+BCL-XL inhibitors (Figure 6E). Collectively, these findings show how the type of anticancer treatment (exemplified here with Decitabine versus MEK_i_+BCL-XL_i_) reshapes the specificity of the immune remodeling in the tumor microenvironment, possibly restricting the tumor immune plasticity to the action of ICIs. Furthermore, the increased heterogeneity observed in tumors treated as well with ICIs may have clinical implications as further complicating prediction of response and pertinent re-adjustment of therapy.

### The immune landscape is differentially reshaped according to the type of treatment in the *Alb-R26*^*Met*^ tumor model

**Figure 7:**
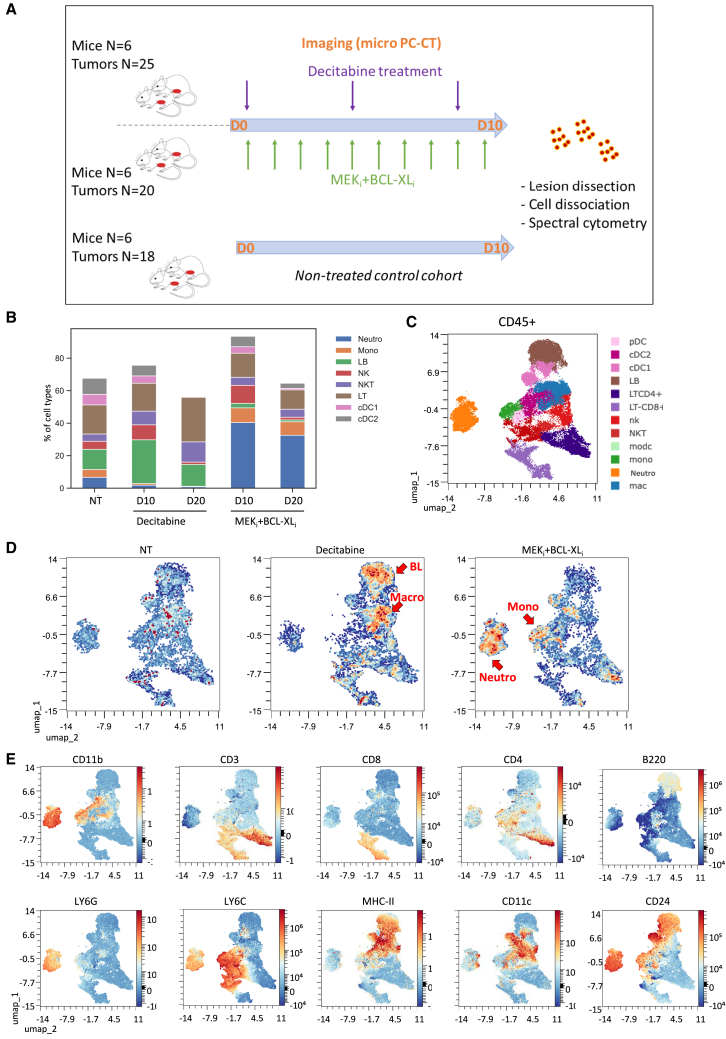
Results from longitudinal *in vivo* imaging of spontaneous tumors in the *Alb-R26*^*Met*^ model to analyze changes in immune cell types after 10 days of treatment (A) Schematic representation of the protocol followed. Two cohorts were treated either with Decitabine or MEK_i_+BCL-XL_i_ during 10 days, compared with untreated controls, before tumor dissociation and spectral flow cytometry analysis. Mice were imaged at D0 and D10. The total number of analysed tumors in each cohort is reported. (B) Histogram showing the percentage of different immune cell populations at D10 and D20 with or without treatment. Histograms at D20, already shown in Figure 4B, are reported here for comparison purposes. (C) Cluster identification results from marker expression levels in tumors analyzed by spectral flow cytometry at D10. The different immune cell clusters are reported on the right. (D) UMAP clustering corresponding to non-treated, Decitabine or MEK_i_+BCL-XL_i_ treated tumors. Note the different “switch on” patterns in relation to the treatment type. Decitabine treated tumors are characterized by increased BL cluster whereas MEK_i_+BCL-XL_i_ treated tumors have increased neutrophil and monocyte clusters. (E) UMAP clustering corresponding to the indicated markers.

The outcomes of anti-PD1+anti-CTLA4 added to Decitabine or MEK_i_+BCL-XL_i_ treatments prompted us to explored whether the immune remodeling according to different anticancer treatments has an impact not only on the composition of distinct CD45^+^ cell types, but also on the proportion of immune cells expressing specific immune-checkpoints. We addressed this question by following the presence of cells expressing specific immune-checkpoints at an intermediary time point (D10; Tables S7 and S8). We reasoned that such setting could provide a better vision of the ongoing remodeling generated by the two distinct treatments we experimentally performed, Decitabine versus MEK+BCL-XL blockage. For this purpose, we designed an antibody panel for a spectral flow cytometry analysis conceived to detect a range of immune-checkpoints (Table S6), also in relation to the bulk RNA-seq results from the *Alb-R26*^*Met*^ tumor model (Figures 2A, 2B, and S2B), while reanalyzing again at D10 the composition of immune cell types in the tumor microenvironment. A new group of *Alb-R26*^*Met*^ tumor-bearing mice was generated to constitute two cohorts treated with Decitabine or MEK_i_+BCL-XL_i_ for ten days and compared with a non-treated cohort as control (Tables S7 and S8), then tumors were dissected and dissociated cells processed for spectral cytometry (Figure 7A). Results show that the differential repartition of the immune cell type compositions in Decitabine versus MEK_i_+BCL-XL_i_ treated tumors starts already at D10 and further progress at D20 (Figures 7B, S7A, and S7B). Indeed, macrophages and BLs are already increased after D10 of Decitabine treatment whereas neutrophils and monocytes are increased upon MEK_i_+BCL-XL_i_ treatment (Figures 7C–7E). Furthermore, the decrease of TLs observed upon MEK_i_+BCL-XL_i_ treatment is predominantly linked to decreased TL CD8^+^, as no differences were found for TL CD4^+^ (Figures S7A and S7B).

**Figure 8:**
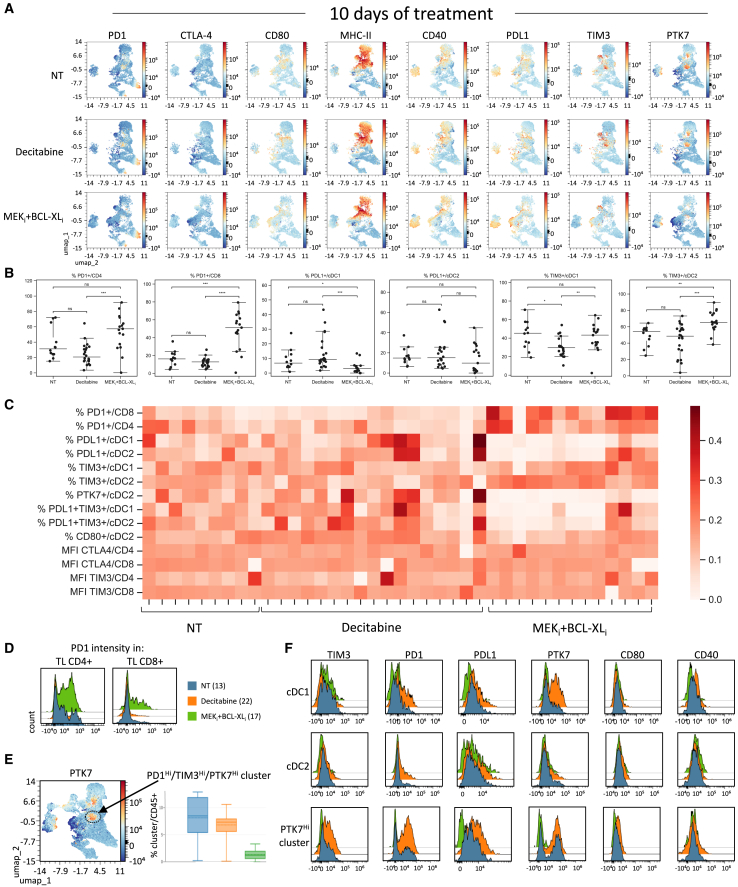
Results from longitudinal *in vivo* imaging of spontaneous tumors in the *Alb-R26*^*Met*^ model to analyze changes in immune checkpoints after 10 days of treatment (A) UMAP clustering corresponding to the indicated markers in non-treated (NT), Decitabine, or MEK_i_+BCL-XL_i_ treated tumors. (B) Dot-plots illustrating spectral flow cytometry analysis of PD1, PDL1, and TIM3 in the indicated immune cell type from non-treated (NT) and treated tumors at D10. Graphs report on the Y axis the percentage of distinct immune cell types indicated on the top of each graph. (C) Normalized heatmap of immune checkpoint expressing cells in non-treated versus treated tumors. (D) Graphs reporting PD1 intensity in TL CD4^+^ and TL CD8^+^ cells in non-treated (NT) and treated tumors. (E) Left: UMAP clustering highlighting a specific cluster characterized by high levels of PD1, TIM3, and PTK7. Right: boxplot reporting the percentage of this specific cluster in the conditions indicated in D (NT: blue; Decitabine: orange; MEK_i_+BCL-XL_i_: green). (F) Graphs reporting the intensity of the indicated immune-checkpoints and of PTK7 in cDC1, cDC2, and PTK7^Hi^ clusters. Statistical analyses were performed using Kruskal Wallis. Levels of significance: ∗*p* ≤ 0.05; ∗∗*p* ≤ 0.01; ∗∗∗*p* ≤ 0.001. Bars represent SEM and notches median levels.

Results revealed as well drastic changes in the proportion of cell types expressing ICIs, particularly in the cohort of MEK_i_+BCL-XL_i_ treated tumors (Figures 8A–8C, S7C–S7E, and S8). Specifically, we found an increase of PD1^+^/CD4 and PD1^+^/CD8 populations in MEK_i_+BCL-XL_i_ treated tumors compared with those treated with Decitabine (Figures 8A–8C, S7C and S7E). The increase of PD1 expression at the surface of TLs is particularly stronger on TL/CD4^+^ compared to TL/CD8^+^ (Figure 8D). Such increase of PD1^+^/TLs in MEK_i_+BCL-XL_i_ treated tumors was also found at the endpoint of the experiment (D20; Figure 4E). We also observed a decrease of PDL1^+^/cDC1 percentage of cells in MEK_i_+BCL-XL_i_ treated tumors, whereas a more heterogeneous repartition among tumors characterises the PDL1^+^/cDC2 population (Figures 8A–8C). No significant changes were detected in the expression levels or percentages of CTLA4^+^/CD4, CTLA4^+^/CD8, or CD80^+^/cDC2 populations (Figures 8B–8C, S7D and S7E). Moreover, we found an increased trend of ICOSL in cDC2 in Decitabine treatment, suggesting that cDC2 infiltrating HCC are mature and can potentially differentiate TLs in TL helper 2 or regulatory TLs (Figures S7D andS7E). We intriguingly found an increase in the percentage of TIM3^+^/cDC1 and TIM3^+^/cDC2 cells in MEK_i_+BCL-XL_i_ treated tumors compared with those treated with Decitabine (Figures 8B and 8C). The TIM3^+^ levels in CD8 and CD4 did not show significant changes with either treatment (Figures S7D, S7E, and S8A). Moreover, tumor compartment (CD45^−^) analyses revealed a decreased trend in cells expressing Galectin9 and GITR in MEK_i_+BCL-XL_i_ treatment, whereas no major ICOSL changes were observed (Figure S8B). Interestingly, we found a cluster of CD45^+^ cells co-expressing high levels of PD1, TIM3, and PTK7, the latter is a pseudokinase overexpressed in HCC,^24^^,^^45^ which was decreased in MEK_i_+BCL-XL_i_ treated tumors compared with untreated or Decitabine-treated tumors (Figures 8E and 8F). Collectively, these results correlate with the effects of PDL1+CTLA4 blockage in relation to Decitabine or MEK_i_+BCL-XL_i_ treatment, pointing to TIM3 as a putative ICI for combinatorial therapy. Altogether, these findings illustrate how different anticancer treatments modify the composition of immune cell types in the tumor microenvironment and the percentage of cells expressing specific immune-checkpoints on their surface.

### Identification of a putative immune signature characterizing tumor treatment response using a machine learning approach

**Figure 9:**
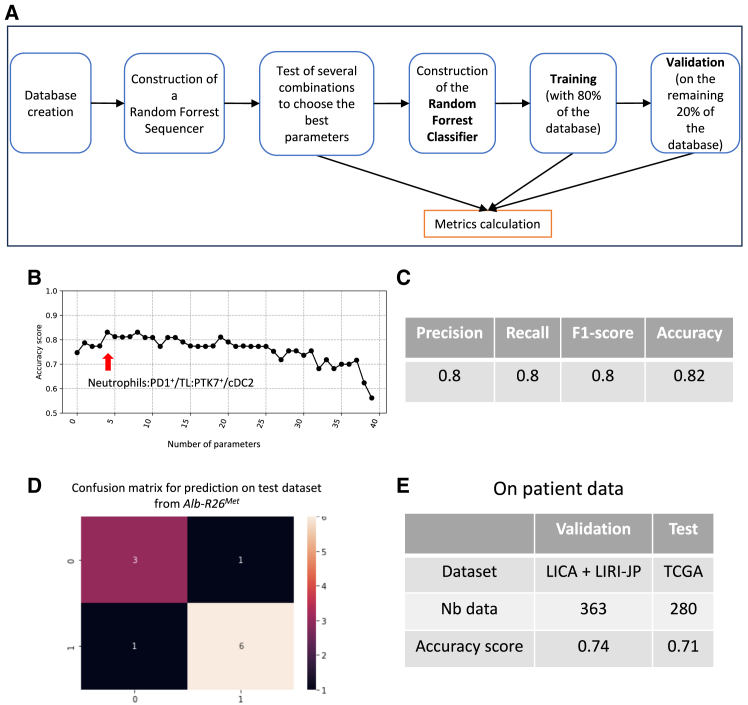
Signature identification using machine learning algorithm based on immune cell subtypes to predict treatment response (A) Schematic representation of the machine learning strategy we used to identify an immune signature predicting the tumor behavior. (B) Graph representing the accuracy score according to the signature and the number of parameters used. The arrow points the dot corresponding to the accuracy of the signature we chose. (C) Table summarizing metrics to assess the random forest classifier on the 20% of our dataset (validation set). The remaining 80% were used to train the model (train set). (D) Confusion matrix of the prediction on the validation set. X axis correspond to the truth and y axis to the prediction. (E) Table with metrics results of the Random Forest build with patient databases. In the first columns, results from the validation of the model and in the second column, results obtained on the test set.

By recapitulating the molecular and immune cell type heterogeneity characterizing HCC patients, the *Alb-R26*^*Met*^ model exemplifies the heterogeneous vulnerability of tumor to distinct treatments, as illustrated here with their behavior as responding or non-responding subtypes following Decitabine or MEK_i_+BCL-XL_i_ treatments. The capability to predict tumor response to treatment remains a major challenge, and currently HCC treatments are used based on the absence of adverse contraindications rather than on reliable parameters to identify putative responding patients. As we did not find any evident immune trait distinguishing responder versus non-responder tumors, we hypothesized that a combinatorial use of distinct parameters might be required. We tested this possibility constituting two groups of tumors (responders vs. non-responders) irrespectively on the type of treatment, to have a sufficient collection of longitudinal imaging and immune datasets for a machine learning approach. We then constructed a random forest (RF) sequencer, which we launched to test several combinations to ultimately select the best parameter set for prediction of tumor response (Figure 9A). We found that an inclusion of several parameters increases the accuracy score up to a certain point, while becoming repetitive or even detrimental for the prediction when too many parameters are included (Figure 9B). This approach brought us to identify a specific combination of three distinct parameters resulting in a pertinent compromise between accuracy and feasibility. This signature takes into consideration the percentage of: (1) neutrophils; (2) PD1^+^/TLs; (3) PTK7^+^/cDC2s. PTK7 is a pseudokinase over-expressed in several solid tumors and hematological malignancies and linked to metastasis, poor prognosis, and resistance.^46^^,^^47^ After constructing the RF classifier considering this signature, we divided all cohorts in training set (80% of the database) and validation set (the remaining 20% of the dataset). Evaluated on the validation set, we found that the accuracy score of this signature is 0.82 (Figure 9C). We then calculated a confusion matrix for the prediction on the validation dataset from the *Alb-R26*^*Met*^ tumors (Figure 9D). Reassuringly, we found that our prediction model is able to predict both tumor classes, the responding and the non-responding subtypes. This is illustrated by the confusion matrix showing an error of one case for both classes, meaning that the training is effective (Figure 9D). To further assess the relevance of the identified signature, we tested its reliability for patient prediction response. First, we used two patient cohorts for which the required datasets are available, the LICA and LIRI-JP, to train (on 80% of the data) and evaluate (on the remaining 20% of the data) our predictive model. This process allowed us to achieve an accuracy score of 0.74 (Figure 9E). Next, we tested the model on another independent dataset (not previously used for training or evaluation of the model), the TCGA cohort, to determine whether the model could be generalized to different datasets. Notably, we obtained an accuracy score of 0.71, which for an RF classifier is rather high to predict the responsiveness of patients based on the signature we found (Figure 9E). Together, these findings exemplify how the combinatorial use of distinct immune cell type populations might offer alternative prediction strategies to discriminate putative responding versus resistant tumors to treatments.

## Discussion

Immunotherapeutic agents have been extensively introduced into clinical trials and for clinical management of several cancer types, with great benefit although only for a proportion of patients. Resistance mechanisms and lack of reliable biomarkers to accurately predict responders, despite significant toxicity in some cases, are among major limitations, restraining their clinical benefit. For years, genomics and immune predictors of patient response have been searched in tumor biopsies before treatment. Although some molecular data are correlative with response, their robustness for prediction remains largely unsatisfactory, particularly for most complex and heterogeneous cancer types. It is the case of HCC, for which after almost two decades of clinical use, RTKi have lost first position to the combined immunotherapy using anti-PDL1 plus either anti-VEGFR or anti-CTLA4. Currently, these treatment options are employed in the absence of adverse contraindications rather than on reliable parameters, which are still not available to select responders. Furthermore, the apparition of severe secondary effects put forward the necessity of finding pertinent combinations and signatures to predict treatment allocation and response. An emerging concept that gains consideration concerns the necessity to also take into account how the immune landscape of tumors with their microenvironment evolves during treatments. Indeed, a growing number of reports provide evidence that anticancer agents can elicit immunostimulatory or immunosuppressive effects in addition to cytostatic/cytotoxic actions on cancer cells.^48^ The overall studies we report here illustrate how distinct anticancer agents, while targeting cancer cells, differentially reshape the tumor immune microenvironment. Several intriguing aspects emerged from our findings.

First, the anticancer treatments we tested superimpose specific immune landscape over the original one. Among changes, MEK_i_+BCL-XL_i_ leads to an upregulation of the percentage of neutrophils and monocytes, whereas Decitabine downregulates both (Figure 4). Changes also include an increased percentage of TLs by Decitabine and decreased BLs by MEK_i_+BCL-XL_i_ (Figure 4). Furthermore, the amplitude in the reduction of cDC1s and cDC2s is significantly different between Decitabine and MEK_i_+BCL-XL_i_ (Figure 4). These effects are likely the consequences on dying cancer cells following treatments and on the direct action of drugs on immune cells, in relation to BCL-XL functions in BLs,^49^ as reported for cDCs by targeting the BCL2 family,^50^ and for CD8^+^ progenitors with an improved responsiveness to anti-PD1 by Decitabine.^51^^,^^52^^,^^53^ Different treatments also have distinct effects on the proportion of cells expressing the immune-checkpoints we tested. For example, PDL1^+^/cDC1s are decreased whereas PD1^+^/CD8s and TIM3^+^/cDC2s are increased in MEK_i_+BCL-XL_i_ treated tumors (Figure 8B). We extensively processed spectral cytometry and imaging data to search parameters correlating with tumor responders versus non-responders. No correlations were found in relation to the percentage of specific immune cell types. Instead, an unbiased analysis of multiple parameters through a machine learning approach identified a signature characterizing tumor response, based on the percentage of neutrophils, PD1^+^/TLs, and PTK7^+^/cDC2s. The robustness of this signature is supported by analyzing datasets from the *Alb-R26*^*Met*^ cancer model and from three HCC patient cohorts for which data could be exploited for this analysis. Additional independent cohorts will strengthen its pertinence to predict response, also in relation to treatment types. Of notice, by deconvoluting GEO: GSE109211 RNA-seq datasets from HCC patient cohorts, we did not observe an immune cell type remodeling in sorafenib treated patients compared to placebo (Figure S9), indicating a differential proficiency of anticancer drugs to influence the tumor immune landscape.

Second, both types of anticancer treatments we tested flatten the heterogeneity present in pre-treated tumors for some immune cell types. Remarkable examples concern the percentage of cDC1s, cDC2s, and NKs following Decitabine or MEK_i_+BCL-XL_i_ treatments, of BLs in MEK_i_+BCL-XL_i_, and of monocyte and neutrophils in Decitabine treated tumors (Figure 4). The possibility of flattening the immune heterogeneity originally present among tumors and of favoring the emergence of an immune-checkpoint vulnerability linked to a specific anticancer drug could guide toward optimizing immunotherapy types. This reinforces the need of a follow-up of patients during treatment using biopsies, with potential tremendous clinical benefit, although clinically challenging.

Third, we found that PDL1+CTLA4 blockage combined with Decitabine increases the number of responding tumors. This may correlate with the increase infiltration of macrophages we observed in Decitabine-treated tumors. Instead, PDL1+CTLA4 blockage does not further improve MEK_i_+BCL-XL_i_ treatment efficiency, rather we observed the emergence of few quiescent/non-responding tumors, otherwise not present in MEK_i_+BCL-XL_i_ alone. These findings are also intriguing considering the increased population of PD1^+^/CD8 TLs we found in MEK_i_+BCL-XL_i_ treated tumors, which has been reported to correlate with exhausted signature and poor clinical outcome in HCC.^54^ We cannot exclude that the moderate effects we observed adding anti-PDL1+anti-CTLA4 might be due to a short time treatment (about 13 days). These outcomes suggest that monitoring distinct cell populations expressing immune-checkpoints during treatments might avoid a non-optimal use of immunotherapeutic agents, which can either do not provide beneficial effects or even turn out to be detrimental for patients, in addition to unnecessary side effects. Instead, we intriguingly found an increased percentage of TIM3^+^/cDCs in MEK_i_+BCL-XL_i_ treated tumors. Future studies using complementary mouse cohorts, ideally based on different liver cancer models, could be designed to explore the possibility of potentiating oncotherapy response, like those elicited by MEK_i_+BCL-XL_i_, blocking TIM3. Of notice, there are currently 104 clinical trials (https://clinicaltrials.gov/search?cond=Cancer&term=tim3), including 7 for liver cancer (https://clinicaltrials.gov/search?cond=liver%20Cancer&term=tim3) on TIM3 blocking agents. Furthermore, in view of the increased PD1^+^/CD8 TL population found in MEK_i_+BCL-XL_i_ treated tumors, it would be also pertinent to assess potential benefit of combined blockage of TIM3 and PD1.

Fourth, the tumor regression triggered by MEK+BCL-XL blockage we reported here using the *Alb-R26*^*Met*^ mice is rather impressive, particularly considering the molecular and immune tumor heterogeneity recapitulated by this cancer model and by the drastically remodeling of the immune profile of treated tumors. As aforementioned, combined blockage of MEK+BCL-XL/BCL2 is currently explored in two clinical trials, for patients with advanced or metastatic solid tumors, and with recurrent ovarian and endometrial cancers. We think that this combinatorial treatment deserves further consideration in additional clinical trials. Our findings highlight the importance to analyze samples during treatment, with a desirable objective of identifying the best immunotherapy to use in combination. Furthermore, it would be necessary to carefully define the most pertinent timing of anticancer and ICI treatment, in view of a recent study showing that PDL1 blockage briefly before MAPK targeting improve antitumor immunity and efficacy in mice.^55^ Finally, it would be important to determine the amplitude of antigens released following MEK_i_+BCL-XL_i_ and whether cancer cells undergo immunogenic cell death, initiating adaptive immune responses, thus potentiating the therapeutic effects of this drug combination.^56^

In conclusion, our studies exemplify how the tumor immune landscape undergo dramatic remodeling during treatments, engaged in distinct immune responses depending on the targeted therapy. We propose that achieving the desired success of immunotherapy in the clinic requires: (1) a dynamic vision of tumors with a follow-up biopsy to adequate the therapy according to their evolution during treatment instead of determining the whole therapeutic program on a static vision of pre-treated tumors; (2) the evaluation on how pertinent biomarkers/signatures evolve over time to accurately allocate patients, to predict their response, and to redirect non-responders to alternative options; (3) a broader vision for selecting the most pertinent ICIs in relation to patient signature and immune profile changes, instead of aligning all patients with a given cancer type to a given ICI, likely ineffective—or even inadequate—for some of them. This would require an intellectual leap to explore the adequateness of alternative procedures in the clinic, benefitting of machine learning approaches to predict signatures.

### Limitations of the study

The use of the *Alb-R26*^*Met*^ model, recapitulating resistance and molecular/temporal heterogeneity of liver cancer patients, strengthens the clinical relevance of outcomes. Nevertheless, findings deserve additional studies testing other anticancer drugs, using larger cohorts of mice, multiple independent experiments, different immune-competent cancer mouse models reproducing immune heterogeneity, and ideally patient biopsies before and after treatments. As tumors in the *Alb-R26*^*Met*^ model are resistant to multiple RTKi used in the clinic for HCC treatment, we could not explore how the immune landscape evolves during tumor regression triggered by RTKi. This topic is clinically relevant, although conditioned by the identification of drugs conferring vulnerability of HCC cells to RTKi, which remains a highly desirable challenge. Still, our results document the possibility of expanding the use of anti-cancer agents, like Decitabine and MEK_i_+BCL-XL_i_ employed for other cancer types, by exploiting their capability to remodel the immune landscape. A further topic that deserves to be explored is whether the switch of immune cell types and immune-checkpoints we report in liver cancer occurs in other cancer types, to design pertinent therapeutic options particularly for patients resistant to available treatments.

## Resource availability

### Lead contact

Requests for further information and resources should be directed to and will be fulfilled by the lead contact, Flavio Maina (flavio.maina@univ-amu.fr).

### Materials availability

This study did not generate new unique reagents.

### Data and code availability

• RNA sequencing raw data are publicly accessible via the NCBI Gene Expression Omnibus (GEO) under accession number GEO: GSE271709. Raw counts for each analyzed sample are reported in Data S2.
• Code and data used for the random forest classifier referring to the Figure 9 can be found on this link: https://gitlab.in2p3.fr/imxgam-public/code-for-iscience-d-24-02353r4.
• Any additional information required to reanalyze the data reported in this paper is available from the lead contact upon request.

## Acknowledgments

These results are in part based upon public data generated by TCGA Research Network: http://cancergenome.nih.gov/or in public repositories including ICGC DataPortal, Firebrowse, and NCBI GEO. We thank: X. Adhoute and E. Soucie for extremely valuable feedback on the manuscript; F. Castagna for precious help on liver dissections and spectral cytometry; Dr. M Heim and colleagues for giving us data access to the EGAS00001005074 HCC patient cohort (HCC-NatCom cohort); all members of our labs for helpful discussions; people at the CRCM and IBDM core facilities for their essential technical support on mouse, microscopy, and flow cytometry; the ICEP histology platform for essential assistance and support; T. Boudier at CENTURI for valuable inputs on data processing. This work was financially supported by GEFLUC—Les Entreprises contre le Cancer, ARC (Association pour la Recherche sur le Cancer; PJA20181208172 and ARCPGA2023010005822_6352), FdF (Fondation de France; 2016_00067080), and by 10.13039/501100006364INCa (2020-11/279/NI-KA) to F.M.; with financial support from ITMO Cancer of Aviesan within the framework of the 2021–2030 Cancer Control Strategy, on funds administered by Inserm (21CD125-00) to F.M. and J.-P.B; from AFEF (Association Française pour l’étude du Foie) and Canceropôle Provence-Alpes-Côte d’Azur—Institut Cancer Immunologie to C.S. This work received support from the French government under the France 2030 investment plan, as part of the Initiative d'Excellence d'Aix-Marseille Université - A∗MIDEX (AMX-19-IET-001). F.M. lab receives funding from Région Provence-Alpes-Côte d’Azur and Canceropôle Provence-Alpes-Côte d’Azur, SATT Sud-Est, and Ruban Rose Avenir. C.S. was supported by a 10.13039/501100002915Fondation pour la Recherche Médicale fellowship. F.C. was supported by the 80❘Prime project DePIcT of the Mission pour les Initiatives Transverses et Interdisciplinaires (MITI) of CNRS. The contribution of the Region Provence-Alpes-Côte d’Azur and of the Aix-Marseille Univ to the CRCM and IBDM animal facility is acknowledged. The development of the PC-CT PIXSCAN-FLI prototype was partly supported by France Life Imaging (grant ANR-11-INBS-44-0006 from the French “Investissement d’Avenir’’ program) and by the 10.13039/501100014182Canceropôle Provence-Alpes-Côte d’Azur. The funders had no role in study design, data collection and analysis, decision to publish, or preparation of the manuscript.

## Author contributions

F.C.: performed *in vivo* studies, imaging analysis and processed data; created algorithm for imaging tumor segmentation, 3D reconstruction, and quantification; contributed to dataset analysis and interpretation; processed tumors for cell dissociation analysis and spectral cytometry data for quantifications and UMAP clustering; constructed random forest classifier for signature prediction; contributed to interpret data; provided inputs on studies and contributed to write the manuscript. C.S.: performed computational work with mouse and human transcriptomics datasets; processed tumors for cell dissociation analysis and spectral cytometry data for quantifications; contributed to interpret data; provided inputs on studies and contributed to write the manuscript. P.M.V.: performed spectral cytometry analysis and data interpretation; provided inputs on studies and on the manuscript. A.E.K.: performed, interpreted, and correlated bioinformatic analyses of mouse and human bulk RNA-seq data. M.M.: performed spectral cytometry analysis and data interpretation; provided inputs on studies. S.R.: contributed to *in vivo* studies and to processed tumors for cell dissociation analysis. M.K.: performed anatomo-pathological analyses of *Alb-R26*^*Met*^ tumors. A.C.: provided inputs for deep learning analysis. J.-P.B.: provided input on studies and on the manuscript. C.M.: supervised and provided input on *in vivo* imaging studies and on the manuscript. Y.B.: supervised and provided input on *in vivo* imaging studies and on the manuscript. F.M.: designed the study, contributed to experimental work, analyzed and interpreted data, ensured financial support, and wrote the manuscript.

## Declaration of interests

The authors declare that they have no competing interest.

## STAR★Methods

### Key resources table

**Table:** 

REAGENT or RESOURCE	SOURCE	IDENTIFIER
**Antibodies**		

anti-human CD11b	BD Biosciences	clone M1/70; RRID: AB_2738276
anti-mouse CD8	BD Biosciences	clone 53–6.7; RRID: AB_2872946
anti-mouse CD88	BD Biosciences	clone 20/70; RRID: AB_2873018
anti-mouse CD24	Miltenyi	clone REA743; RRID: AB_2656545
anti-mouse NK1.1	BD Biosciences	clone PK136; RRID: AB_2738617
anti-mouse CD45	Biolegend	clone 30-F11; RRID: AB_2564590
anti-mouse MHCII	Invitrogen	clone M5/114.15.2; RRID: AB_465232
anti-mouse Ly6C	Biolegend	clone HK1.4; RRID: AB_2566577
anti-mouse CD11c	Biolegend	clone N418; RRID: AB_493568
anti-mouse Ly6G	Biolegend	clone 1A8; RRID: AB_10640452
anti-mouse CD3	Miltenyi	clone REA641; RRID: AB_2751847
anti-human CD274/PDL1	Biolegend	clone 10F.9G2; RRID: AB_2875856
anti-mouse CD80	BD Biosciences	clone 16-10A1; RRID: AB_2870102
anti-mouse CD40	BD Biosciences	clone 3/23; RRID: AB_2734767
anti-mouse CD279/PD1	Biolegend	clone 29F.1A12; RRID: AB_2562616
anti-mouse CD276/B7-H3	Biolegend	clone MIH35; RRID: AB_2800638
anti-mouse CD152/CTLA-4	Invitrogen	clone UC10-4B9; RRID: AB_465879
anti-mouse CD366/TIM3	Biolegend	clone RMT3-23; RRID: AB_2922462
anti-mouse/human B220	Biolegend	clone RA3-6B2; RRID: AB_312996
anti-mouse/human PTK7	Home made	clone 1H6
anti-mouse/human B220	Biolegend	clone RA3-6B2; RRID: AB_2563491
anti-mouse CD278/ICOS	Miltenyi	clone 7E.17G9; RRID: AB_2660680
anti-mouse LGAL9	Biolegend	clone RG9-35; RRID: AB_2562296
anti-mouse GITRL	Biolegend	clone YGL 386; RRID: AB_2287690
anti-mouse CD275/ICOSL	Miltenyi	clone REA990; RRID: AB_2727548
anti-mouse F4/80	BD Biosciences	clone T45-2342; RRID: AB_2873657

**Chemicals, peptides, and recombinant proteins**		

Decitabine	TargetMol	T1508
MEK inhibitor (Trametinib)	TargetMol	T2125
BCL-XL inhibitor (ABT-737)	TargetMol	T2099
anti-PDL1	Bio X Cell	BX-BE0101
anti-CTLA4	Bio X Cell	BX-BE0131

**Deposited data**		

Bulk RNA-Seq of *Alb-R26*^*Met*^ mice	GEO	GSE271709

**Experimental models: Organisms/strains**		

*Alb-R26*^*Met*^ mice	in house colony	

**Software and algorithms**		

CIBERSORT	TIMER 2.0 portal	http://timer.cistrome.org/
Python 3.6	Anaconda	https://anaconda.org/anaconda/python
ITK Snap Software	ITK-SNAP 4.2.2	http://www.itksnap.org/pmwiki/pmwiki.php
SpectroFlo Software	CytekBio	https://cytekbio.com/pages/spectro-flo
OMIQ software	Dotmatics	https://www.omiq.ai/
Graphpad software	Prism v.8	https://www.graphpad.com/

### Experimental model and study participant details

#### Ethics statement for *in vivo* animal studies

All procedures involving the use of animals were performed in accordance with the European Community Council Directive of 22 September 2010 on the protection of animals used for experimental purposes (2010/63/EU). The experimental protocols were carried out in compliance with institutional Ethical Committee guidelines for animal research (comité d’éthique pour l’expérimentation animale – Comité d’éthique de Marseille) and in compliance with French law, under an agreement number E1305521, Ministère de l’Enseignement Supérieur de la Recherche et de l’Innovation. Mice were kept in a dedicated pathogen-free facility, with a light/dark cycle, and in cages with an enriched environment. Mice received Safe Complete Care Competence (SAFE A04) as complete aliment *ad libitum* and were housed in environmentally enriched cages under pathogen-free conditions. The project authorization of Maina laboratory relevant to this study is APAFIS #8214–2016121417291352.v5, delivered by the “Ministère de l’Enseignement Supérieur, de la Recherche et de l’Innovation”.

#### *Alb-R26*^*Met*^ mice generation

The generation of the *R26*^*stopMet*^ mice (international nomenclature *Gt(ROSA)26Sor*^*tm1(Actb-Met)Fmai*^) carrying a conditional mouse-human chimeric *Met* transgene inserted at the *Rosa26* locus has been previously reported.^25^^,^^57^^,^^58^ In the *R26*^*stopMet*^ model, slightly enhanced wild-type MET levels is achieved following removal of the stop cassette using the Cre recombinase.^26^ In the *Alb-R26*^*Met*^ mice, with increased MET levels in the liver, tumors spontaneously form over time. Tumors recapitulate the most aggressive HCC patient subtype defined as “proliferative-progenitor”, the primary resistance to drugs used in clinic, the molecular heterogeneity of patients, and the temporal heterogeneity of tumor onset.^26^ The *Alb-R26*^*Met*^ HCC, as HCC patient subsets, is characterized by a striking enrichment in genes that are simultaneously overexpressed and hypermethylated in gene body CpG islands.^24^ The mouse line expressing Cre recombinase under the *Alb* promoter (*B6.Cg-Tg(Alb-cre)21Mgn/J*) was obtained from the Jackson Laboratory. *Alb-R26*^*Met*^ mice were generated by crossing the *R26*^*stopMet*^ (in C57BL/6JRj strain) and *Alb-Cre* (in 129S2/SvPasOrlRj strain) mice. Consequently, tumors formed in F1 C57:129Sv *Alb-R26*^*Met*^ mice, genotyped by PCR analysis of genomic DNA as previously reported.^25^^,^^26^ Only male mice, carrying both *Alb-Cre* and the *R26*^*stopMet*^ mutations (named as *Alb-R26*^*Met*^ mice) were used in these studies. The age of most mice ranges between 50 and 70 weeks old (only two mice were 81 and 105 weeks old), in relation to the heterogeneity of tumor onset, which is another unique feature of the *Alb-R26*^*Met*^ liver cancer model. The age of each mouse used in these studies is reported in Tables S3, S7, and S8.

### Method details

Methods concerning *in vivo* experimentation are detailed below and in figure legends following recommendations of ARRIVE guidelines for transparency and reproducibility (https://arriveguidelines.org/sites/arrive/files/documents/ARRIVE%20guidelines%202.0%20-%20English.pdf).

#### Histo-pathology of Alb*-R26*^*Met*^ HCC mouse model

*Alb-R26*^*Met*^ were dissected, and tumors were processed as FFPE (formalin-fixed paraffin embedded), snap frozen and bulk transcriptomics samples. Microtome sections of FFPE tumors were stained with Hematoxylin and a pathologist from ICEP platform was asked to diagnose and characterize the grade and immune infiltration of each sample. The sections of liver samples (*n* = 20; 3 healthy livers; 14 HCC tumors; and 3 tumor adjacent liver tissue) were evaluated by a pathologist using light microscope (Leica DM LB2). Pathologic grading of tumors was made by using two different systems. The four-tiered modified Edmondson–Steiner system, and the three-tiered WHO grading system (detailed information in Table S1). Additionally, we used nuclear and nucleolar gradings to have more histologic clues about the tumor samples. Mitotic counts for tumor samples were made on the most mitotically active areas on 10 consecutive HPF (High power field; 400x). For the samples which include both HCC and non-tumoral liver, mitotic count and the other scorings were made on HCC areas, not on non-tumoral areas.

#### Bulk RNA-seq analysis of the *Alb-R26*^*Met*^ mouse model

Bulk RNA-seq studies were performed by following a procedure we previously reported.^24^ Briefly, mRNA was extracted with Qiagen kit following manufacturer instructions from a total of six livers (from mice that did not develop tumors) and twelve advanced tumors from *Alb-R26*^*Met*^ mice (Table S3 and Data S2). 500ng of total RNA per sample was diluted in RNase- DNase- and protease-free molecular grade water (concentration >20 ng/μL; maximum volume 20 μL). RNA quality was verified with bioanalyzer and purity considered as valid when OD_260/280_ was between 1.8 and 2.2, with RIN values equal or superior to 8. Samples were submitted to ICM for sequencing with Illumina Novaseq X technology (purification of poly-A containing mRNA molecules; 40 million reads/sample, 100 reads each, paired end). Reads were quality-checked using FastQC and aligned to mouse reference genome GRCm38 (mm10). Quantification was performed using Salmon 1.10.2, and the generated counts were pre-processed and normalized using Transcripts Per Million (TPM). Principal Component Analysis (PCA) was performed using the PCA function from the FactoMineR R package. Data S2 reports RNA-seq data as raw counts for each analyzed sample.

#### Analysis of publicly available human RNA-seq data

The human RNA-seq and clinical data from LIHC-TCGA were available through the UCSC Xenabrowserportal (https://xenabrowser.net/). LIHC-TCGA includes 50 normal livers and 372 primary tumor samples, from which only 371 have survival information. Both LICA-FR (161 tumor samples) and LIRI-JP (202 normal liver and 243 tumor samples) are available through the ICGC Data Portal (https://dcc.icgc.org/). HCC-NatCom patient cohort^59^ was accessed through the EGA repository (European Genome-phenome Archive; EGAS00001005074) after contacting the corresponding author and obtaining an agreement of a data use policy in order to protect patient confidentiality. STORM trial (GEO: GSE109211; patients receiving as adjuvant treatment either placebo (73 patients) or sorafenib (67 patients) were downloaded from the NCBI GEO data portal (https://www.ncbi.nlm.nih.gov/gds).

#### Estimation of immune cell composition by deconvolution

Deconvolution of available bulk RNA-seq (from our lab for the mouse cohort and from public databases for patient cohorts) to estimate the percentage of immune cell subtypes in samples was performed using CIBERSORT^60^ and MCPcounter, an online open tool available at TIMER 2.0 portal through http://timer.cistrome.org/. Data were uploaded to the webpage and the parameters were set to either mouse/human HCC.

#### Correlation analyses of human and mouse data

Bar-plots were used to visualize the proportion of patients with deregulated gene expression within the four independent HCC patient cohorts (TCGA, LIRI-JP, LICA-FR, HCC-NatCom). For each cohort, log_2_ fold change (log_2_FC) was calculated by comparing gene expression in each tumor sample to the mean gene expression of corresponding control samples from the same cohort (or with the GTEx normal liver samples in the case of the LICA cohort as lacking corresponding control samples). The bar-plots represent the proportion of patients in each cohort in which a given gene is deregulated. Plots were created using the R ggplot2 package, with the x axis representing genes and the y axis representing the proportion of deregulated patients.

To assess the relationship between gene expression profiles in *Alb-R26*^*Met*^ tumors and HCC patient cohorts, we performed Pearson correlation analysis. For each gene, the average expression across samples was calculated separately for each transcriptomic dataset. Pearson correlation coefficients (R) and associated *p*-values were computed to quantify the strength and significance of the correlations.

Scatterplots were generated to visualize the correlation between datasets, with a regression line fitted to the data. Plots were created using the R ggplot2 package, with each point representing the mean expression of a gene. The regression line and confidence intervals were displayed to highlight the relationship between the *Alb-R26*^*Met*^ transcriptome and each HCC cohort.

Principal Component Analysis (PCA) was performed to visualize variance in gene expression and immune profiles across different sample groups. PCA was applied to 12 *Alb-R26*^*Met*^ tumors and 6 control liver samples using log-transformed and scaled gene expression values as input. Additionally, immune cell type scores obtained from deconvolution analysis of bulk RNA-seq data from mouse tumors and HCC patient cohorts were used for PCA to compare immune cell composition across samples. PCA was also conducted on immune profile estimations for each *Alb-R26*^*Met*^ tumor to explore immune heterogeneity within the tumor samples. For all PCA analyses, the first two principal components were computed and visualized in a 2D space. Plots were generated using the R ggplot2 package, with samples distinguished by color or shape to represent their respective groups. Axes were annotated with the percentage of variance explained by each principal component.

#### *In vivo* imaging with the PIXSCAN-FLI

PIXSCAN-FLI is a photon counting micro-computed tomography (PC-CT) prototype scanner that we previously reported.^33^ Briefly, it is made of three main blocks whose characteristics are: a) the X-ray source block embedding an X-ray tube Microfocus L12161-07 (Hamamatsu Photonics K.K., Japan) with a tungsten anode; b) the animal block embedding a rotative motor for performing tomographic scans that can be also translated along the three axes; c) the X-ray hybrid pixel camera comprising more than 500.000, 500 μm thick, 130 μm × 130 μm square silicon pixels. To perform imaging, we used a specific barium contrast agent, the ExiTronNano 12000 (Miltenyi Biotec©), which is up-taken by macrophages and liver resident Kupffer cells, therefore leading to highly contrasted liver (and spleen) thanks to barium radio-opacity. Mice were anesthetized with gas anesthesia Vetflurane to process the scans. They received a radiation dose of 110 mGy per scan. Imaging data acquisition and processing were performed following previously described procedure.^33^ Tumor volumes were calculated on the basis of tumor segmentations. These were carried out in two steps. Firstly, a pre-segmentation was performed by deep learning. Briefly, a neuronal network with a U-Net architecture^61^ was built and trained on scans and tumor segmentations already achieved with PIXSCAN-FLI.^33^ As a result, the U-Net architecture provides 3D segmentations of the tumors in the reconstructed volumes. The difficulty to detect small tumors and to remain reliable when acquisition parameters are modified necessitated to check manually and complete the final segmentations using the ITK Snap software. Subsequently, the number of segmented voxels in a tumor multiplied by their size gives the tumor volume.

#### Drug administration protocol

*Alb-R26*^*Met*^ mouse males at the age supposed to start developing tumors (after 40 weeks of age) were imaged by PC-CT. According to imaging outcomes, only mice with tumors were processed to constitute the indicated cohorts. Tumor-bearing mice were used to constitute the distinct groups reported in the schemes, distributing them randomly in relation to the size and number of tumors, to achieve comparable groups of individuals. For the following imaging analyses, blinding was not performed as the operator needed to associate a mouse ID to the imaging system, to compare subsequent images of the same mouse during treatments, and for the treatment administrations. As the main objective of our study was to uncover the immune composition of tumors either untreated or treated with distinct treatments, which cannot be predicted or biased by looking at a mouse ID, we considered that blinding was not necessary.

For the first experiment, a cohort was treated for 10 or 20 days, every four days by intraperitoneal injection with Decitabine (5 mg/kg; TargetMol) resuspended in DMSO and diluted in Cremophor and NaCl 0.9%. Another cohort was treated for 10 or 20 days, every day by intraperitoneal injection with Trametinib (1 mg/kg, MEK inhibitor; TargetMol) plus ABT-737 (30 mg/kg, BCL-XL inhibitor; TargetMol), both diluted in Cremophore and NaCl 0.9%.

For the second experiment, two other cohorts were treated for 20 days with the same protocol as described above (with Decitabine or MEK+BCL-XL inhibitors) starting the co-administration of immunotherapeutic agents at day 7 of treatment. Immunotherapy consisted in the intraperitoneal injection every four days with two immune checkpoint blocking antibodies: an anti-PDL1 (7.5 mg/kg; Bio X Cell ref. BX-BE0101) and an anti-CTLA4 (7.5 mg/kg; Bio X Cell ref. BX-BE0131), both diluted in NaCl 0.9%.

Mouse body weight was monitored 3 times per week and injections were adapted according to the animal ethical regulations (a reduction of 20% of the mice weight was considered as an ethical point to pause the treatment, and in case of no recovery euthanasia was considered). Imaging was performed every 10 days. Livers and tumors were dissected; a piece was immediately frozen in dry ice for RT-qPCR studies and another used for cell dissociation followed by spectral cytometry analysis.

The endpoint of *in vivo* experiments were either 10 days or 20 days after the first day of drug administration. However, the longitudinal imaging follow-up allowed us to refine the methodology by evaluating if we reached the maximal ethical tumor volume permitted before the end of the experiment (in our case mice did not reach this ethical point before the experimentally established endpoint).

#### Cell dissociation for spectral flow cytometry

Dissected tumors, livers (from mice without tumors) or tumor-adjacent livers (liver tissue in the surrounding area of a tumor) were placed in a 2 mL Eppendorf tube for cell dissociation with 1 mL of an enzymatic solution composed by collagenase IV (0.75 mg/mL; Sigma Aldrich, ref. C1538-500MG) and DNAse I (25 μg/mL; Sigma Aldric, ref. 11284932001) diluted in CO_2_-independent medium (ThermoFisher Scientific, ref. 11580536) and supplemented with 5% FBS and penicillin/streptomycin. Tumors were incubated 15–20 min in a water bath at 37°C, pipetting up and down every 5 min to facilitate cell dissociation. The solution with dissociated cells was then filtered through 70 μm nylon strainers into a new 2 mL tube, and the filter was washed with 1 mL of FACS buffer (48.8 mL of PBS, 1 mL of FBS, 200 μL of EDTA 0.5M pH8). Dissociated cells were incubated 5 min on ice and centrifuged 5 min at 1000 rpm 4°C. The cell pellet was resuspended in 1 mL of ACK red blood cell lysis buffer (A1049201; ThermoFisher Scientific), incubated 1 min on ice, then centrifuged for 5 min at 1000 rpm 4°C. The cell pellet was then resuspended in 500 μL of FACS buffer. Cell count and viability were verified with trypan blue using a Biorad Cell Counter (TC20), and samples maintained on ice till processed by spectral cytometry analysis.

#### Spectral cytometry analysis

For all single cell suspension from dissociated tumors, cells were washed and filtered, and Fc receptors were blocked (anti-CD16/CD32, Invitrogen). Cells were then incubated with the corresponding antibody panel (Table S6) for 15 min at 4°C in FACS buffer. Cells were washed twice and resuspended in FACS buffer plus 5 ng/mL of DAPI. Samples were acquired in the spectral cytometer Aurora from Cytek, with the 5L 16UV-16V-14B-10YG-8R configuration, and using spectro-flow software. Supervised and unsupervised analyses were performed using OMIQ software. Clustering of filtered CD45^+^ cells was performed using FlowSOM method and UMAP was used to visualize the tumor immune compartment.

#### Machine learning algorithm

The random forest (RF) algorithm (Figure 9) was generated in Python (anaconda3 version, scikit-learn library). Data were first pre-processed by a standard normalization in order to have the mean equal to zero in the database. Then, an RF sequencer was applied to find the best signature based on a combination of distinct immune cells populations. With the best combination, an RF classifier was created and trained with 80% of the data. The 20% remaining data were used to validate the training. For patients’ cohorts, LICA+LIRI-JP databases were used for training and test, whereas TCGA was used for the test. The accuracy score was used to describe the performances of the model. F1-score and confused matrices were also calculated to determine the performances.

### Quantification and statistical analysis

To calculate the sample size needed for the *in vivo* experiment to obtain significative results while respecting the 3R rule, we used an adequate method of calculation: the Resource Equation Method.^62^^,^^63^ This method allows to calculate the degree of freedom of one-way ANOVA test, given by the *DF* (between-subject error): *DF* *=* *N–k =* *kn–k* = *k* (*n–1*) where *N* is the total number of mice; *k* is the number of groups; and *n* is the number of mice per group. The *DF* must be in the range of 10–20: if *DF* < 10 it means that more mice are required to increase the chance of obtaining significant results; if 10 < *DF* < 20 the sample size is considered as adequate; if *DF* > 20 means that increasing more the number of mice per group will not increase the chances of obtaining significant results. In the case of group comparison with repeated measures (tumor volumes at different time points in a dynamic study), we need to calculate the *DF* for between-subject error *DF*1 and the within-subject error *DF*2: *DF= between-subject error DF1 + within-subject error DF2.* The *DF* between subject error *DF*1 is calculated as previously, and the within-subject error *DF* is calculated as follows: *DF* *=* *k*(*n–1*)+*k*(*n–1*)(*r–1*) *=* *k*(*n–1*)(*1*+*r–1*) *=* *kr*(*n–1*), where *N* is the total number of subjects; *k* is the number of groups; *n* is the number of subjects per group; and *r* is the number of repeated measurements.

All data were analyzed using GraphPad Prism software (version-7 and version-8) or Python (anaconda3 version). Normality of the samples was assessed using Shapiro-Kolmogorov test. Statistically, significant differences between 2 groups of samples were estimated by applying an unpaired Student’s t test, or one-way ANOVA (for more than two groups of samples) followed by multiple comparison Dunnet’s test (all samples compared to control), Tukey’s test (all samples compared) to data showing normal distribution. For non-parametric samples, either Mann-Whitney test (2 groups) or Krustall-Wallis (more than 2 groups) was used. Results are expressed as the mean ± standard error of the mean (SEM). Each dot in graphs corresponds to an independent analyzed sample. Statistical significance (*p*-values) was defined as not significant (ns): *p* > 0.05; ∗: *p* < 0.05; ∗∗: *p* < 0.01; ∗∗∗: *p* < 0.001. Significance is indicated in the Figures.

## Supplemental information

Document S1. Figures S1–S9 and Tables S1–S8

Data S1. Percentage of HCC patients from the indicated cohorts with alterations of genes found in Alb-R26Met transposon studies:27 overexpression of 29 predicted oncogenes, downregulation of 84 predicted tumor suppressors, and altered expression of 137 deregulated genes

Data S2. Table reporting RNA-seq data as raw counts for each analyzed sample

Data S3. Table reporting the expression levels of immune checkpoints from transcriptomic studies in Alb-R26Met livers and tumors
