# Supplementary material for: Tracing specificity of immune landscape remodeling associated with distinct anticancer treatments
Source: iScience. 2025 Feb 20;28(3):112071. doi: 10.1016/j.isci.2025.112071 (PMC11930375; doi:10.1016/j.isci.2025.112071)
Supplement: Document S1. Figures S1–S9 and Tables S1–S8 [file mmc1.pdf]

iScience, Volume *28*

Supplemental information

Tracing specificity of immune landscape remodeling associated with distinct anticancer treatments

Floriane Cannet, Célia Sequera, Paula Michea Veloso, Abdessamad El Kaoutari, Melissa Methia, Sylvie Richelme, Muge Kaya, Afef Cherni, Mathieu Dupont, Jean-Paul Borg, Christian Morel, Yannick Boursier, and Flavio Maina

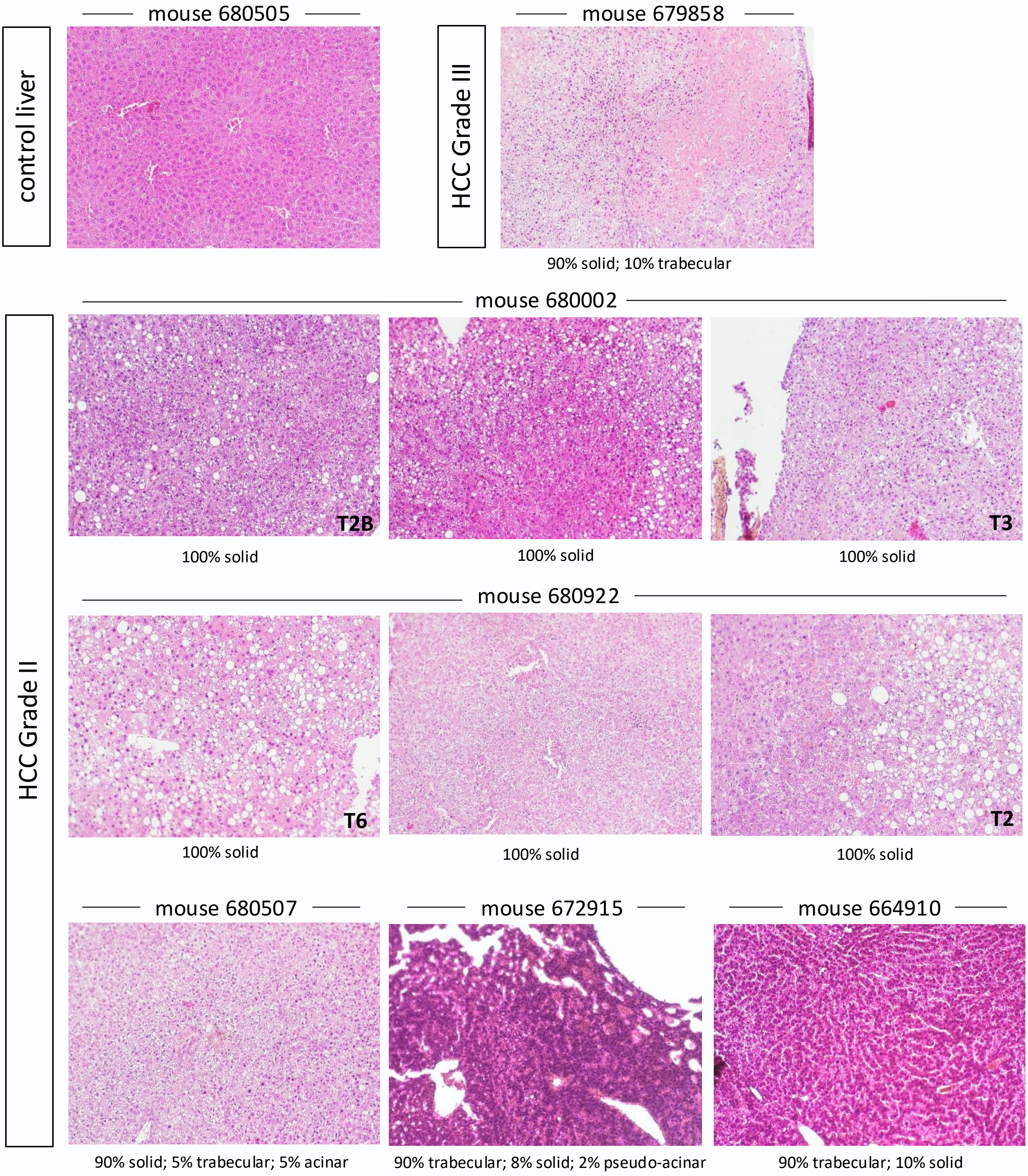
mouse 680505
control liver
mouse 679858
HCC Grade III
90% solid; 10% trabecular
mouse 680002
HCC Grade II
T2B
100% solid
100% solid
T3
100% solid
mouse 680922
T6
100% solid
100% solid
T2
100% solid
mouse 680507
mouse 672915
mouse 664910
90% solid; 5% trabecular; 5% acinar
90% trabecular; 8% solid; 2% pseudo-acinar
90% trabecular; 10% solid

Figure S1

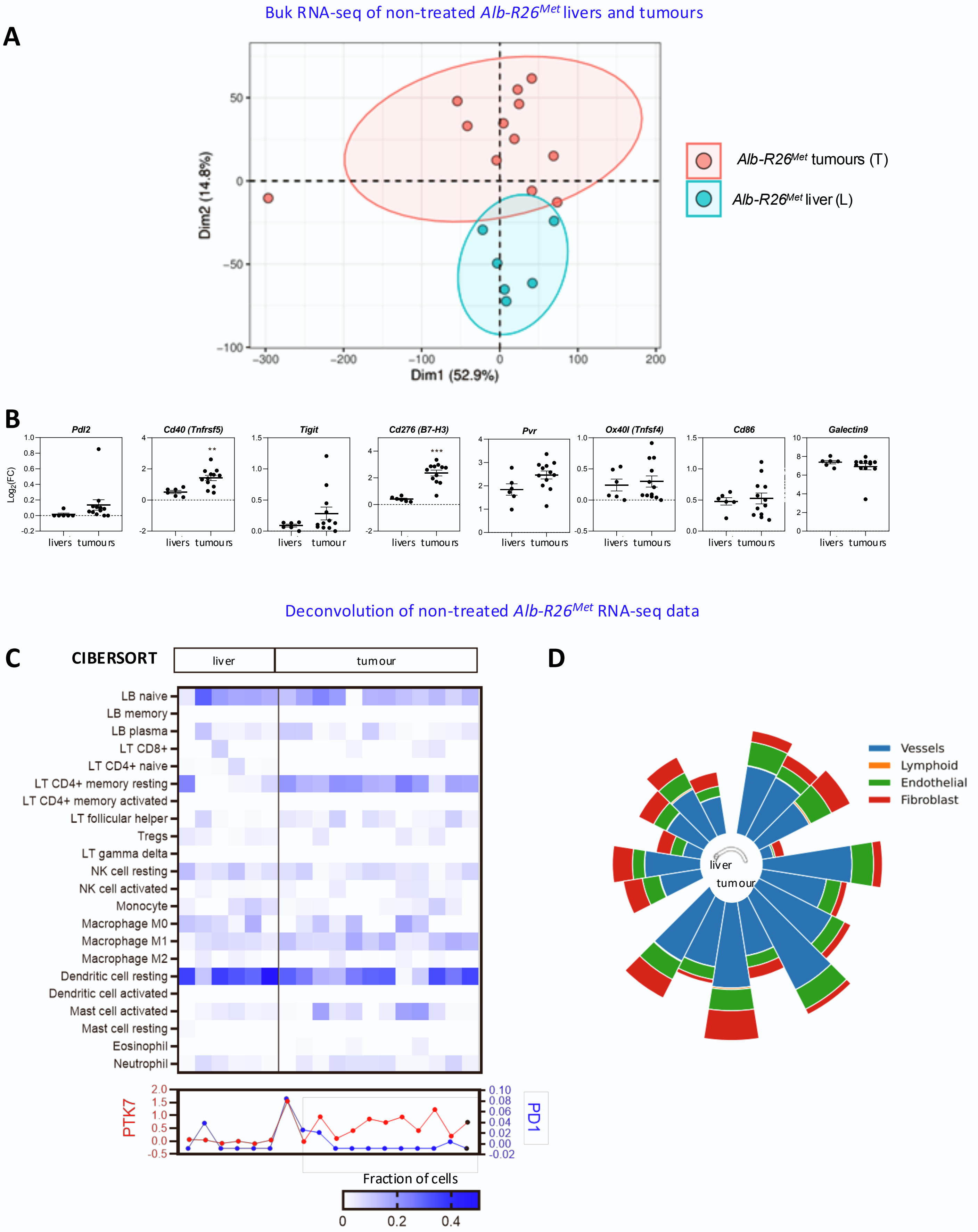

Buk RNA-seq of non-treated Alb-R26Met livers and tumours
A
Dim2 (14.8%)
Dim1 (52.9%)
Alb-R26Met tumours (T)
Alb-R26Met liver (L)
B
Pdl2
Cd40 (Tnfrsf5)
Tigit
Cd276 (B7-H3)
Pvr
Ox40l (Tnfsf4)
Cd86
Galectin9
Log2(FC)
livers tumours
Deconvolution of non-treated Alb-R26Met RNA-seq data
C
CIBERSORT
liver
tumour
LB naive
LB memory
LB plasma
LT CD8+
LT CD4+ naive
LT CD4+ memory resting
LT CD4+ memory activated
LT follicular helper
Tregs
LT gamma delta
NK cell resting
NK cell activated
Monocyte
Macrophage M0
Macrophage M1
Macrophage M2
Dendritic cell resting
Dendritic cell activated
Mast cell activated
Mast cell resting
Eosinophil
Neutrophil
PTK7
PD1
Fraction of cells
D
Vessels
Lymphoid
Endothelial
Fibroblast
liver
tumour

Figure S2

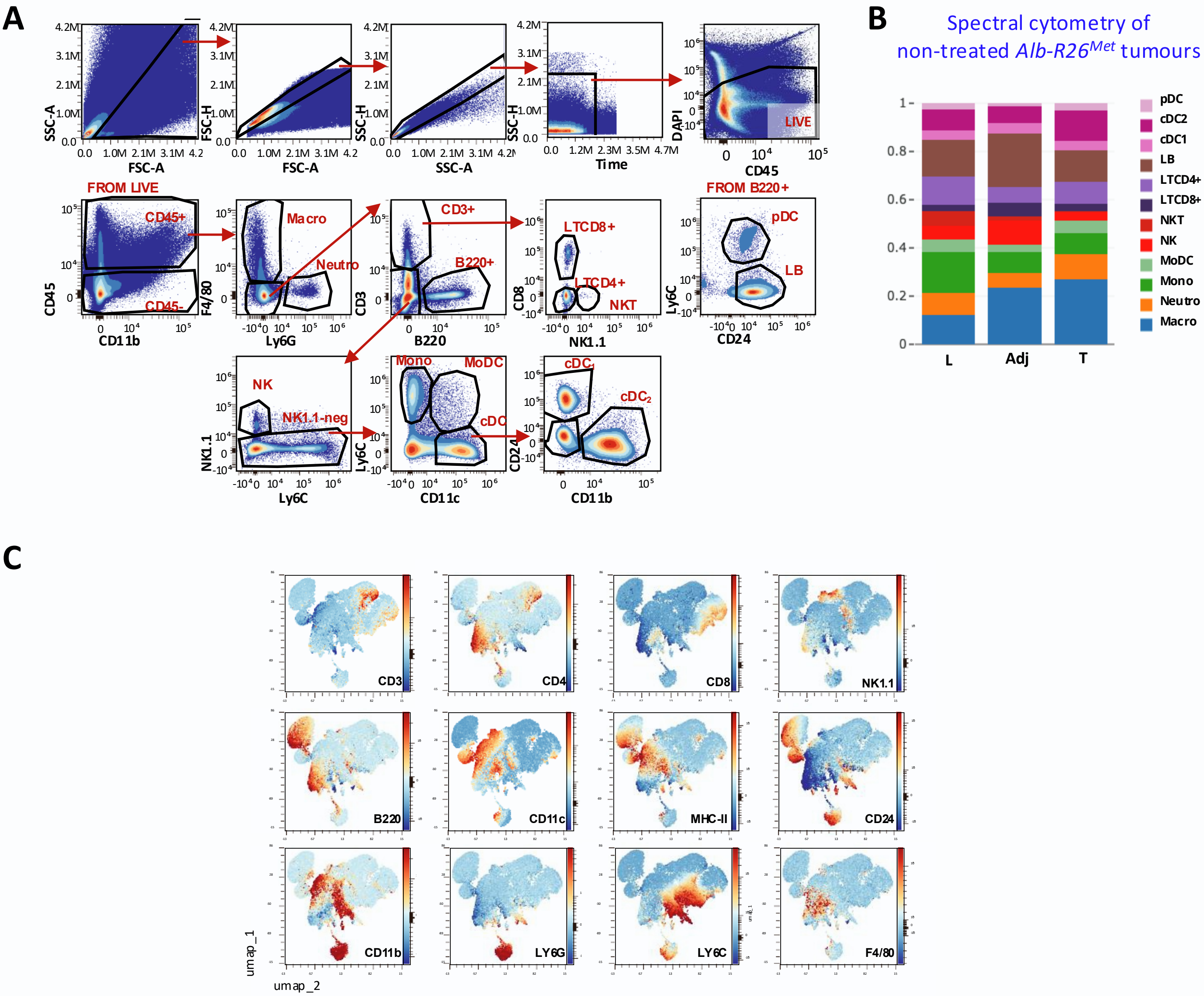

A
B
C
SSC-A
FSC-A
FSC-H
SSC-H
SSC-A
Time
DAPI
CD45
LIVE
FROM LIVE
CD45+
CD45-
CD11b
Macro
Neutro
F4/80
Ly6G
CD3+
B220+
CD3
B220
LTCD8+
LTCD4+
NKT
CD8
NK1.1
FROM B220+
pDC
LB
Ly6C
CD24
NK
NK1.1-neg
Mono
MoDC
cDC
CD11c
cDC1
cDC2
Spectral cytometry of
non-treated *Alb-R26^Met* tumours
pDC
cDC2
cDC1
LB
LTCD4+
LTCD8+
NKT
NK
MoDC
Mono
Neutro
Macro
L
Adj
T
CD3
CD4
CD8
NK1.1
B220
CD11c
MHC-II
CD24
CD11b
LY6G
LY6C
F4/80
umap_1
umap_2

Figure S3

A

	RECIST protocol	Our protocol
Lesions analysis	Disease : sum of the lesions	Each lesions independently
Measure	Sum of the longest lesions diameters	Volume of the lesions
Classification	**Complete response** = disappearance of all targets lesions<br>**Partial response** = 30% decrease<br>**Progressive disease** = 20% increase<br>**Stable disease** = other cases	**Regressive lesion** = 20% decrease<br>**Evolutive lesion** = 20% increase<br>**Stable lesion** = other cases

B

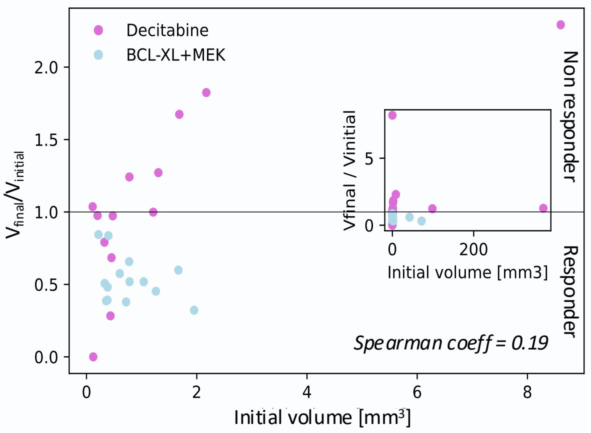

C

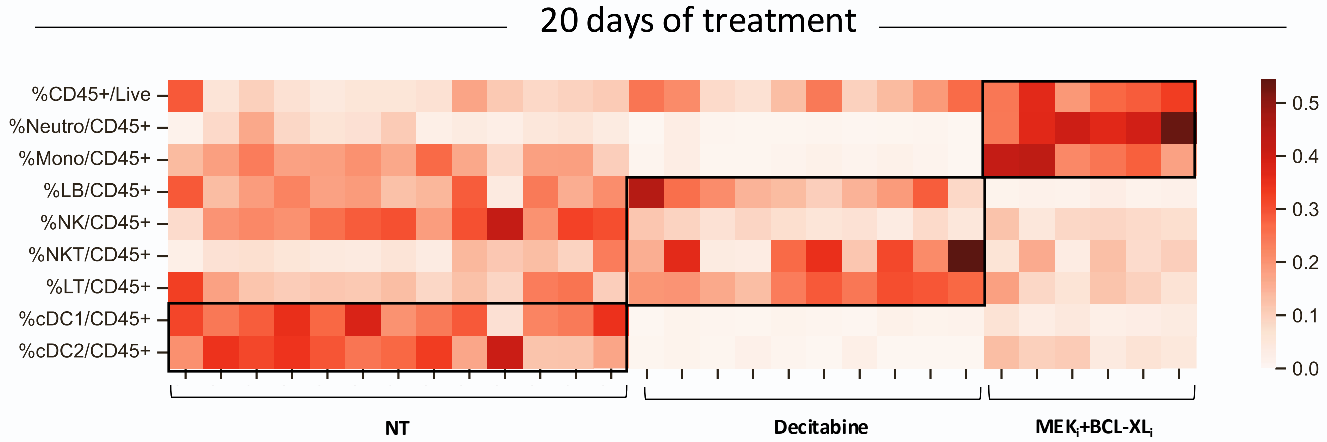

Figure S4

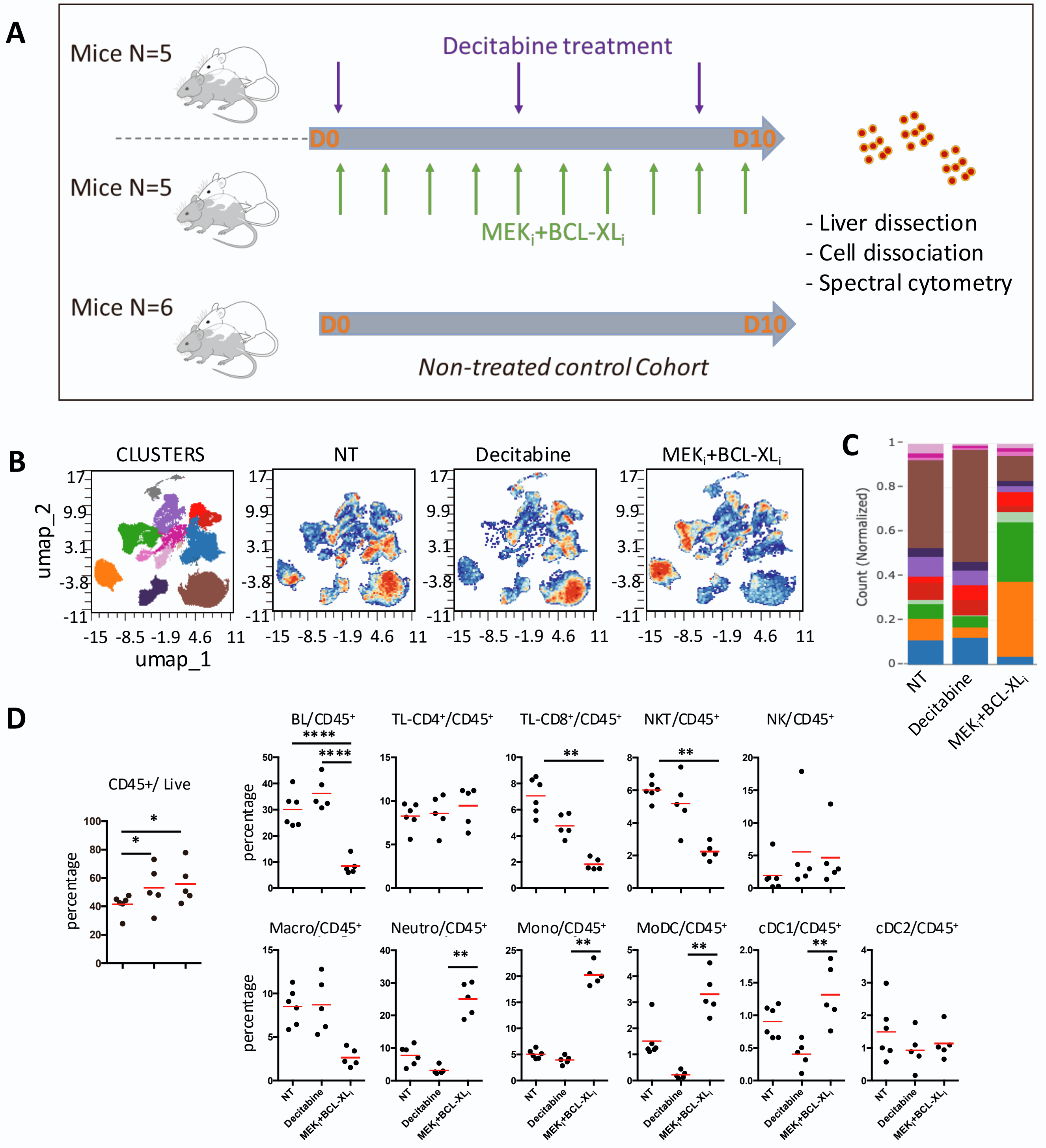
A
Mice N=5
Decitabine treatment
D0
D10
Mice N=5
MEKi+BCL-XLi
- Liver dissection
- Cell dissociation
- Spectral cytometry
Mice N=6
D0
D10
Non-treated control Cohort
B
CLUSTERS
NT
Decitabine
MEKi+BCL-XLi
umap_2
umap_1
C
Count (Normalized)
NT
Decitabine
MEKi+BCL-XLi
D
CD45+/ Live
percentage
BL/CD45+
TL-CD4+/CD45+
TL-CD8+/CD45+
NKT/CD45+
NK/CD45+
Macro/CD45+
Neutro/CD45+
Mono/CD45+
MoDC/CD45+
cDC1/CD45+
cDC2/CD45+
NT
Decitabine
MEKi+BCL-XLi

Figure S5

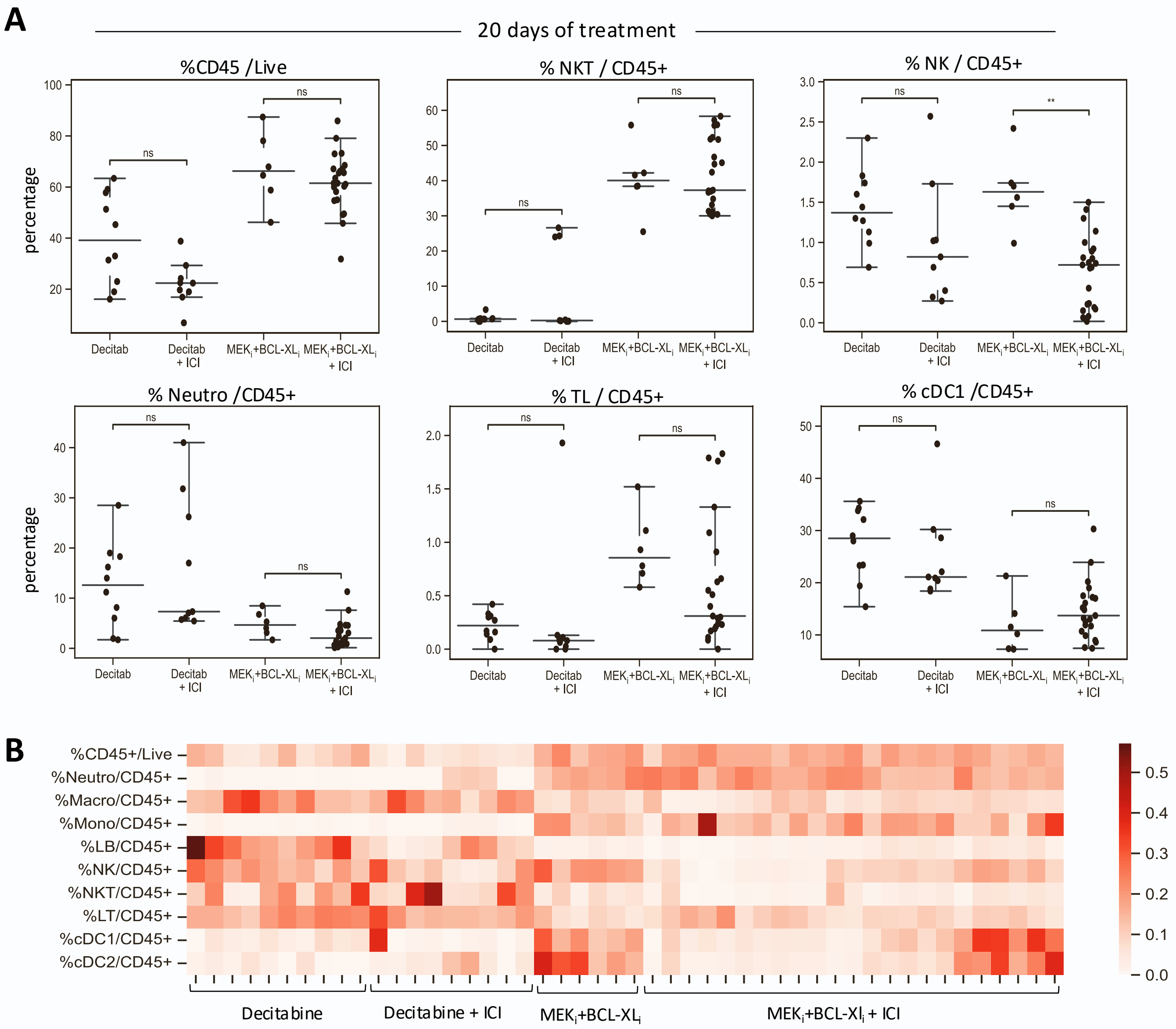
A
20 days of treatment
%CD45 /Live
% NKT / CD45+
% NK / CD45+
% Neutro /CD45+
% TL / CD45+
% cDC1 /CD45+
percentage
ns
**
Decitab
Decitab + ICI
MEKi+BCL-XLi
MEKi+BCL-XLi + ICI
B
%CD45+/Live
%Neutro/CD45+
%Macro/CD45+
%Mono/CD45+
%LB/CD45+
%NK/CD45+
%NKT/CD45+
%LT/CD45+
%cDC1/CD45+
%cDC2/CD45+
Decitabine
Decitabine + ICI
MEKi+BCL-XLi
MEKi+BCL-Xli + ICI

Figure S6

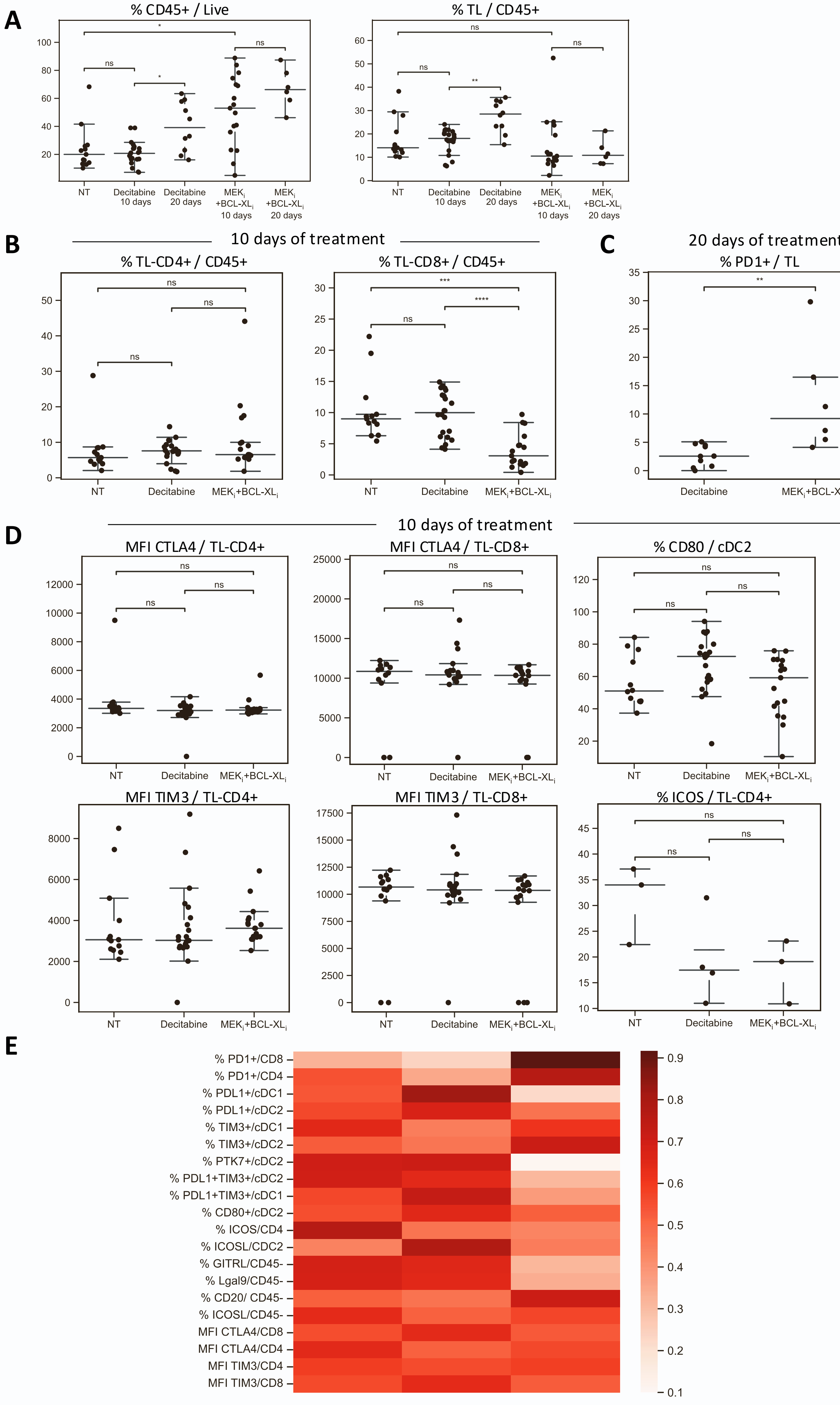
A
% CD45+ / Live
% TL / CD45+
NT
Decitabine 10 days
Decitabine 20 days
MEK$_i$ +BCL-XL$_i$ 10 days
MEK$_i$ +BCL-XL$_i$ 20 days
B
10 days of treatment
% TL-CD4+ / CD45+
% TL-CD8+ / CD45+
C
20 days of treatment
% PD1+ / TL
Decitabine
MEK$_i$+BCL-XL$_i$
D
10 days of treatment
MFI CTLA4 / TL-CD4+
MFI CTLA4 / TL-CD8+
% CD80 / cDC2
MFI TIM3 / TL-CD4+
MFI TIM3 / TL-CD8+
% ICOS / TL-CD4+
E
% PD1+/CD8
% PD1+/CD4
% PDL1+/cDC1
% PDL1+/cDC2
% TIM3+/cDC1
% TIM3+/cDC2
% PTK7+/cDC2
% PDL1+TIM3+/cDC2
% PDL1+TIM3+/cDC1
% CD80+/cDC2
% ICOS/CD4
% ICOSL/CDC2
% GITRL/CD45-
% Lgal9/CD45-
% CD20/ CD45-
% ICOSL/CD45-
MFI CTLA4/CD8
MFI CTLA4/CD4
MFI TIM3/CD4
MFI TIM3/CD8

Figure S7

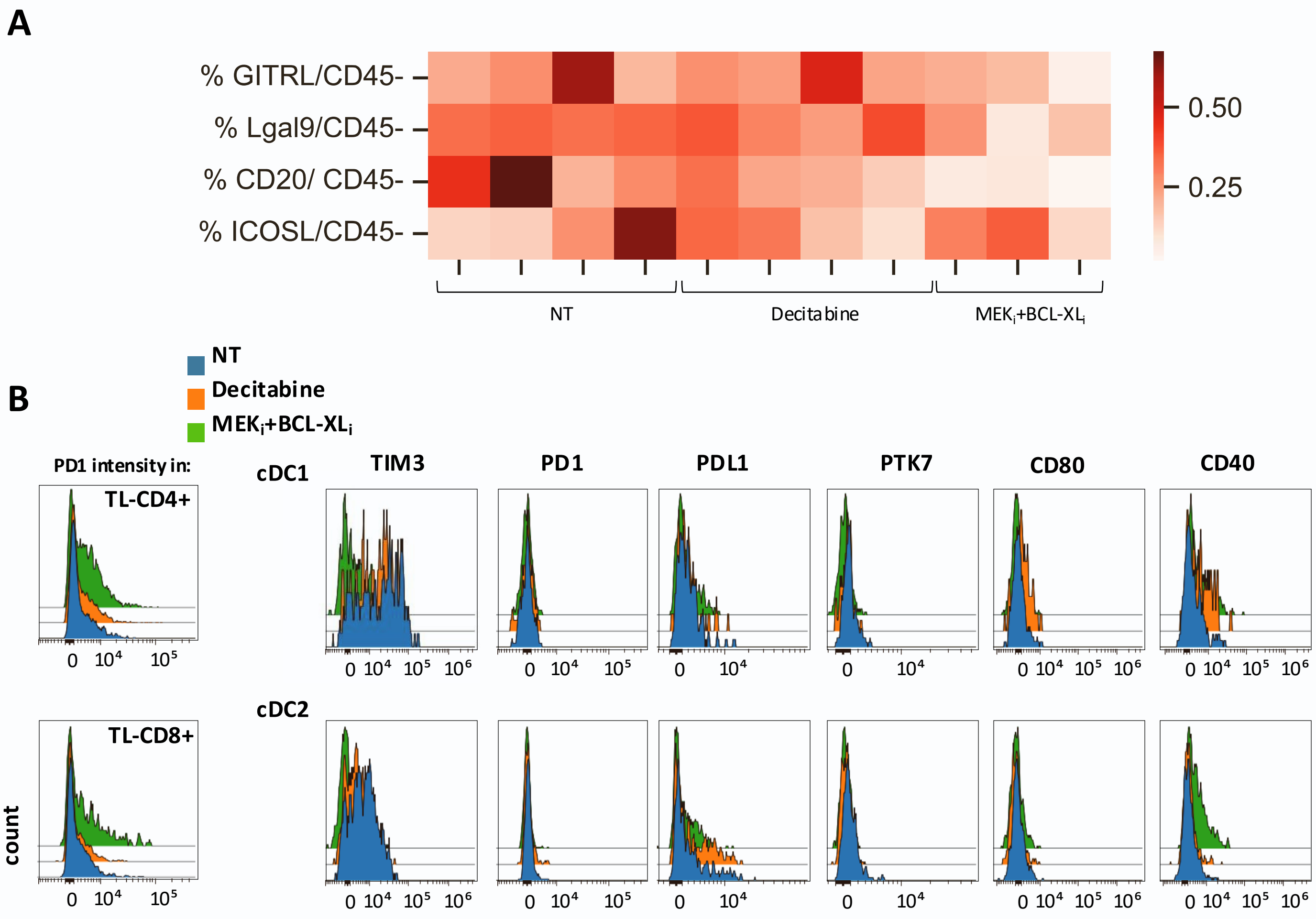

A
% GITRL/CD45-
% Lgal9/CD45-
% CD20/ CD45-
% ICOSL/CD45-
0.50
0.25
NT
Decitabine
MEKi+BCL-XLi
B
NT
Decitabine
MEKi+BCL-XLi
PD1 intensity in:
TL-CD4+
TL-CD8+
count
cDC1
cDC2
TIM3
PD1
PDL1
PTK7
CD80
CD40

Figure S8

A

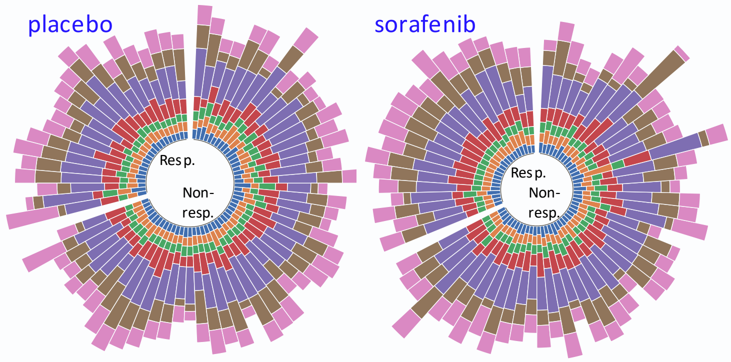

Deconvolution of HCC patients (GSE109211) treated with:
placebo
sorafenib
Resp.
Non-resp.

B

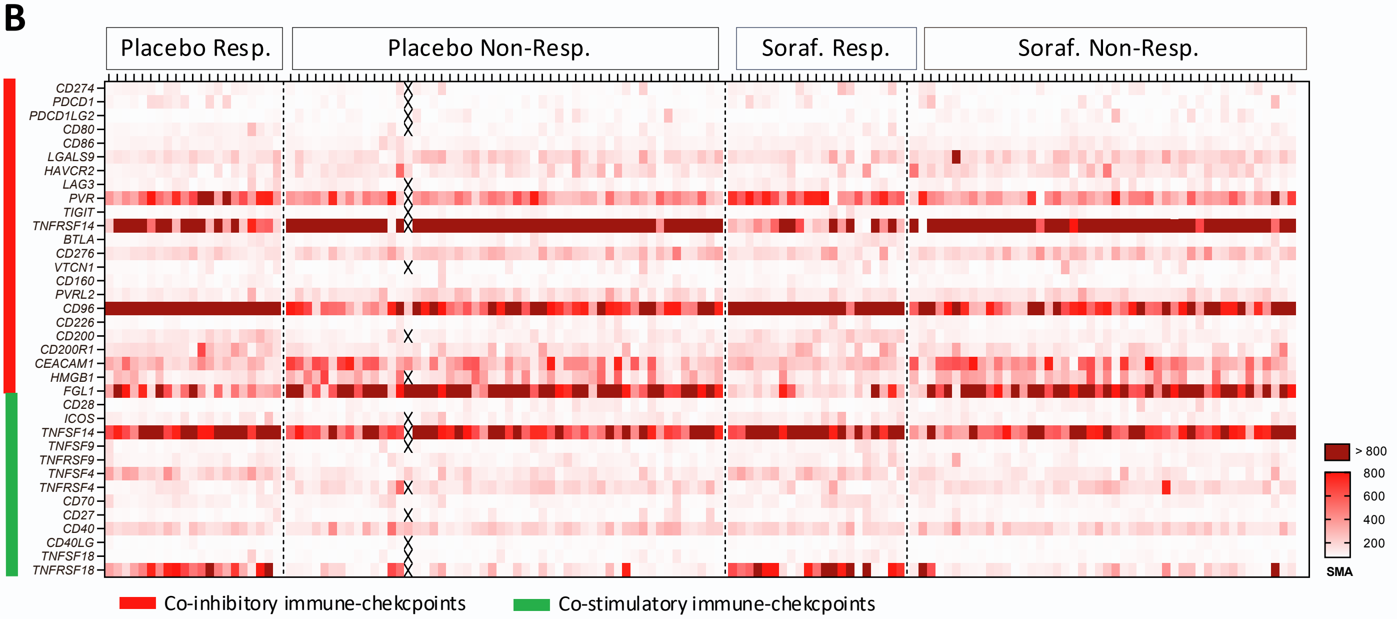

Placebo Resp.
Placebo Non-Resp.
Soraf. Resp.
Soraf. Non-Resp.
CD274
PDCD1
PDCD1LG2
CD80
CD86
LGALS9
HAVCR2
LAG3
PVR
TIGIT
TNFRSF14
BTLA
CD276
VTCN1
CD160
PVRL2
CD96
CD226
CD200
CD200R1
CEACAM1
HMGB1
FGL1
CD28
ICOS
TNFSF14
TNFSF9
TNFRSF9
TNFSF4
TNFRSF4
CD70
CD27
CD40
CD40LG
TNFSF18
TNFRSF18
> 800
800
600
400
200
SMA
Co-inhibitory immune-chekcpoints
Co-stimulatory immune-chekcpoints

Figure S9

SUPPLEMENTARY FIGURE LEGENDS

Figure S1. Pathological HES slides of FFPE tumours from *Alb-R26^{Met}* liver cancer model, related to Fig. 1. HES slides and pathological diagnosis confirmed the HCC entity of tumours and showed that tumours coming from the *Alb-R26^{Met}* liver cancer model are heterogeneous, with different tumour grade and growth pattern.

Figure S2. The immune cell population composition in the *Alb-R26^{Met}* liver cancer model recapitulates patient heterogeneity, related to Fig. 1 and 2. (A) Principal Component analysis (PCA) of samples from bulk RNA-seq in *Alb-R26^{Met}* non-tumoral livers (*n*=6) and advanced tumours (*n*=12). (B) Dot-plots of samples from bulk RNA-seq in *Alb-R26^{Met}* non-tumoral livers (*n*=6) and advanced tumours (*n*=12). Results report the expression levels of the indicated immune-checkpoints (log_2FC). (C) Heatmap of deconvoluted immune composition using CIBERSORT of RNA-seq data from *Alb-R26^{Met}* samples reported in Fig. 2A. Curves under the heatmap indicate the corresponding mRNA levels of PD1 (blue) and PTK7 (red), in relation to the predictive signature we identified (reported in Fig. 9). (D) Circular plots illustrating the heterogeneity of tumour and stromal cell populations based on the deconvolution of *Alb-R26^{Met}* RNA-seq data. Statistical analyses were performed using Kruskal Wallis. Levels of significance: *p ≤ 0.05; ***p ≤ 0.001. Bars represent SEM and notches median levels.

Figure S3. Spectral cytometry analysis of the immune cell population composition in the *Alb-R26^{Met}* liver cancer model, related to Fig. 2. (A) Gating strategy used for spectral flow cytometry analysis to identify the immune cell populations. (B) Histogram showing the count of different immune cell populations from total cells in *Alb-R26^{Met}* control livers, tumour-adjacent livers, and untreated tumours. (C) Scatterplot (coloured-continuous) of UMAP from

Fig. 2E, indicating the corresponding marker of liver, adjacent-tumour and tumours from *Alb-R26^{Met}* mice.

Figure S4. Evaluation of tumour behaviour in *Alb-R26^{Met}* mice treated with Decitabine or MEK+BCL-XL blockage related to Fig. 3 and 4. (A) Comparative table reporting the similarities and the implementations between mRECIST and our protocol to classify tumours as regressive, evolutive, or stable. (B) Graph reporting the tumour reduction after 20 days of treatment, expressed as a ratio between the final and the initial volume. Results show no correlation between the initial size of the tumour and its response. The small square reports all tumours including those with a very big volume. Spearman score is equal to 0.19, indicating no significance. (C) Dot-plots reporting the percentage of NKTs in tumours either non-treated or treated with Decitabine or MEK_i+BCL-XL_i, at D20.

Figure S5. Results from not-bearing control mice to determine the immune landscape in the liver after 10 days of treatment, related to Fig. 5 and 6. (A) Scheme representing the protocol used for this experiment. (B) Single cell unsupervised analysis of spectral cytometry data showing UMAP of $CD45^+$ cluster cells (left) and density-plot of the corresponding tissue (right). (C) Abundance of immune cell population as normalized cell type count from total cells. (D) Quantification of the percentage of $CD45^+$ cells among total live cells and indicated immune cell population among $CD45^+$.

Figure S6. Spectral flow cytometry analysis reveals distinct immune remodelling of the tumoral microenvironment triggered by Decitabine versus MEK+BCL-XL blockage in the *Alb-R26^{Met}* tumours, related to Fig. 5 and 6. (A) Dot-plots reporting immune cell populations in tumours non-treated, treated with Decitabine or MEK_i+BCL-XL_i, with or without immunotherapy at D20. Note the specific change in the immune cell composition linked to

each treatment type. Graphs report on the Y axis the percentage of distinct immune cell types indicated on the top of each graph. (B) Normalized heatmap of cell populations in non-treated versus treated tumours. Statistical analyses were performed using Kruskal-Wallis and Spearman. Levels of significance: *p ≤ 0.05; **p ≤ 0.01; ***p ≤ 0.001. Bars represent SEM and notches median levels. NT: untreated tumours.

Figure S7. Results from longitudinal in vivo imaging of spontaneous tumours in the *Alb-R26^{Met}* HCC mouse model to assess immune checkpoints response after 10 days of treatment, related to Fig. 7 and 8. (A) Dot-plots illustrating spectral flow cytometry analysis of the percentage of CD45^{+} over total live cells and of TLs at D10 and D20. (B-D) Dot-plots reporting percentage of the indicated immune cell populations in non-treated and treated tumours at D10. Graphs report on the Y axis the percentage of distinct immune cell types indicated on the top of each graph. (E) Normalized heatmap of cell populations in non-treated versus treated tumours for each cohort. Kruskal-Wallis. Levels of significance: *p ≤ 0.05; **p ≤ 0.01; ***p ≤ 0.001. Bars represent SEM and notches median levels. NT: untreated tumours.

Figure S8. Expression of distinct immune-checkpoints in CD45-negative untreated and treated *Alb-R26^{Met}* tumours, and TLs and cDCs from treated non-tumour bearing mice, related to Fig. 7 and 8. (A) Normalized heatmap illustrating the percentage of CD45-negative cell populations in non-treated versus treated tumours expressing the indicated immune-checkpoints analysed by spectral flow cytometry. (B) Histograms showing the expression of indicated marker as MFI at the surface of TL-CD4^{+}, TL-CD8^{+}, cDC1, and cDC2. Overlay of non-treated (blue), Decitabine-treated (orange), and MEK$_{i}$+BCL-XL$_{i}$-treated (green) control mice.

Figure S9. The tumoral immune cell type composition and the immune-checkpoint mRNA levels in the GSE109211 patient cohort, related to the discussion section. STORM cohort of

HCC patients (GSE109211), treated with either sorafenib or placebo as adjuvant after resection, segregated into responders or non-responders according to the disease-free survival as cut-off. (A) Circular plots illustrating the heterogeneity of immune cells population based on deconvoluted immune composition using CIBERSORT of RNA-seq data from GSE109211 samples. Results showed an inter-sample heterogeneity both in responders and non-responders. No striking difference on the immune cell profiles between placebo and Sorafenib groups was observed, exemplifying the need of biomarkers/signatures for patient prediction response. (B) Transcriptomic analysis of bulk RNA-seq data from HCC patients (GSE109211), reporting the expression levels of the indicated immune-checkpoints. Patients are organized according to treatment type (placebo and sorafenib) and response (responding and non-responding HCC patients). Of interest, the heatmap representation illustrates "switch on/switch off" patterns of immune-checkpoints common in both treatment groups and in both responders and non-responders. Nevertheless, there are specific patterns that cluster together responders and non-responders, irrespectively on the treatment type (placebo and sorafenib). These observations suggest that responder patients may have a similar expression pattern of some immune-checkpoints, regardless of the treatment type. For example, in both responder groups we observed high levels of *TNFRSF18*, *CD96,* and *TNFS14*. Instead, both non-responder groups are characterised by increased levels of *CECAM1*, *HMGB1*, *FLG1*, and *TNFRSF14.* We also found very subtle difference in *PDL1(CD274)/PD1(PDCD1)* expression levels in responders versus non-responders. This supports the relevance of widening the range of therapeutic agents to consider for HCC treatment to improve its clinical management. The STORM data were obtained using a microarray approach rather than a bulk transcriptomic. Therefore, we could not test properly our signature as it was unable to classify patients correctly.

Table S1, related to Fig. 1. Pathological analysis of HES tumour and liver samples from the *$AlbR26^{Met}$* mice.

Sample no	Pathologic diagnosis	Tumor grade (WHO 2019)	Tumor grade (modified Edmonson-Steiner Grading System)	Histologic subtype (WHO 2019)	Nuclear Grade	Nucleolar Grade	Growth pattern (trabecular/ pseudoacinar/solid/...)	Cytologic atypia (minimal/moderate/marked)	Existance of nodule-in_nodule growth	Vascular invasion	Note primary tumor	Small cell change	Large cell change	Mitotic count/10HPF	Existance of necrosis	Percentage of necrosis (%)	neutrophile count/10HPF	neutrophile localisation	lymphocyte count /10HPF	lymphocyte localisation	eosonophil count/10HPF	eosinophil localisation	Kupffer cell /HPF hot spot	percentage of Kupffer cells/ total liver parenchyme	Kupffer cell localisation	existance of necroinflammatory focus	total no of necroinflammatory focus / x100 magnification (hot spot)	Existance of fatty change	Percentage of fatty change (%)	Benign lesions	Precursor lesions
680 505 HL1	nontumoral	NA	NA	NA	NA	NA	NA	NA	NA	NA	NA	non	non	0	non	NA	13	sinusoidal (5) ; NIF (8)	7	portal tracts (3); NIF (3); sinusoidal (2); parenchymal (5)	0	NA	55	20	Zone 1>zone 2> zone 3	oui	7	oui	5	non	non
679 980 HL	nontumoral	NA	NA	NA	NA	NA	NA	NA	NA	NA	NA	non	non	0	non	NA	10	sinusoidal (10)	10	portal (4), perisinusoidal (6)	0	NA	30	20	Zone 1>zone 2> zone 4	non	NA	oui	2	non	non
680 505 HL2	nontumoral	NA	NA	NA	NA	NA	NA	NA	NA	NA	NA	non	non	0	non	NA	7	inusoidal (4), NIF(3	6	parencymal (3), NIF (3)	0	NA	25	15	no specific distribution	oui	2	oui	20	non	non
680 002 T2b	HCC	Well differentiated	Grade II	conventional (no special subtype)	Grade II	Grade III (nucleoli visible at x100 magnification)	solid	minimal	non	non	no non-tumoral liver	non	non	0	non	NA	10	sinusoidal (10) (subcapsular zone dominancy)	12	parenchymal (5) subcapsular zone; vessel wall (2), parenchymal (5)	0	NA	15	10	no specific distribution	non	NA	oui	25	non	non
680 002	HCC	Well differentiated	Grade II	conventional (no special subtype)	Grade II	Grade III (nucleoli visible at x100 magnification)	solid	minimal	non	non	nontumoral liver tissue transition zone detected	non	non	2	non	NA	18	sinusoidal (3) ; NIF (5); thrombotic perivascular (10)	17	parenchymal (3); NIF (5); thrombotic perivascular (7), portal tract (2)	0	NA	13	10	perivascular	oui	3	oui	30	non	non
680 002 T3	HCC	Well differentiated	Grade II	conventional (no special subtype)	Grade II	Grade III (nucleoli visible at x100 magnification)	solid	minimal	non	non	nontumoral liver tissue transition zone detected	non	non	0	non	NA	16	sinusoidal (13), portal tract (3)	25	parenchymal (15), portal tract (10)	0	NA	20	13	no specific distribution	non	NA	oui	30	non	non
680 922 T7	HCC	Well differentiated	Grade II	conventional (no special subtype)	Grade II	Grade III (nucleoli visible at x100 magnification)	solid	minimal	non	non	nontumoral liver tissue transition zone detected	non	non	0	non	NA	15	sinusoidal (15)	12	parenchymal (12) perisinusoidal	0	NA	20	10	perivascular	non	NA	oui	55	non	non
680 507 T1	HCC	Well differentiated	Grade II	conventional (no special subtype)	Grade II	Grade III (nucleoli visible at x100 magnification)	solid (90%), trabecular (5%), acinar (5%)	minimal	non	non	nontumoral liver tissue transition zone detected	non	non	9	non	NA	7	sinusoidal (7)	10	parenchymal (8), sinusoidal (2)	0	NA	15	10	no specific distribution	non	NA	oui	20	non	non
679 858 T	HCC	Moderately differentiated	Grade III	conventional (no special subtype)	Grade II	Grade II (nucleoli visible at x100 magnification)	solid (90%), trabecular (10%)	moderate	non	non	nontumoral liver tissue transition zone detected	non	non	3	oui	10	16	sinusoidal (6), 10 (necrotic area)	10	parenchymal (5), necrotic area (5)	0	NA	20	10	no specific distribution	non	NA	oui	25	non	non
680 922 T4	HCC	Well differentiated	Grade II	conventional (no special subtype)	Grade II	Grade III (nucleoli visible at x100 magnification)	solid	minimal	non	non	nontumoral liver tissue transition zone detected	non	non	2	non	NA	18	sinusoidal (6), NIF (12)	15	parenchymal (5), NIF (10)	0	NA	20	10	no specific distribution	oui	8	oui	10	non	non
679 922 T6	HCC	Well differentiated	Grade II	conventional (no special subtype)	Grade II	Grade III (nucleoli visible at x100 magnification)	solid	minimal	non	non	no non-tumoral liver	non	non	2	oui	1	14	vessel wall (3), parenchymal (3), sinusoidal (8)	10	parenchymal (7), vessel wall (3)	0	NA	15	10	no specific distribution	oui	2	oui	40	non	non
679 959 T3	HCC	Well differentiated	Grade II	conventional (no special subtype)	Grade II	Grade III (nucleoli visible at x100 magnification)	solid	minimal	non	non	nontumoral liver tissue transition zone detected	non	non	1	non	NA	29	sinusoidal	9	parenchymal (4), stromal (5)	0	NA	20	10	no specific distribution	oui	NA	oui	20	non	non
679 922 T7	HCC	Well differentiated	Grade II	conventional (no special subtype)	Grade II	Grade III (nucleoli visible at x100 magnification)	solid	minimal	non	non	nontumoral liver tissue transition zone detected	non	non	0	non	NA	7	sinusoidal	22	parenchymal (14), portal tracts (8), 200/lymphoid aggregate in portal tract)	0	NA	15	10	no specific distribution	non	NA	oui	60	non	non
673 836 T1 PFA 24	HCC	Well differentiated	Grade II	conventional (no special subtype)	Grade II	Grade II (nucleoli visible at x200 magnification)	solid	minimal	non	non	nontumoral liver tissue transition zone detected	non	non	2	non	NA	4	sinusoidal	2	parenchymal	0	NA	15	10	no specific distribution	non	NA	oui	10	non	non
672 915 T1 20	HCC	Well differentiated	Grade II	conventional (no special subtype)	Grade II	Grade II (nucleoli visible at x200 magnification)	trabecular 90%, solid 8%, pseudoacinar 2%	minimal	non	non	very narrow nontumoral liver tissue transition zone detected	non	non	9	non	NA	25	sinusoidal	35	parenchymal (30), perisinusoidal(5)	0	NA	15/HPF	11	no specific distribution	non	NA	oui	5	non	non
664 909 TA 20 HCC	HCC	Well differentiated	Grade II	conventional (no special subtype)	Grade II	Grade II (nucleoli visible at x200 magnification)	solid	minimal	non	non	no non-tumoral liver	non	non	1	non	NA	10	sinusoidal	5	parenchymal	0	NA	20/HPF	15	no specific distribution	non	NA	oui	20	non	non
664 910 T2 HCC 7	HCC	Well differentiated	Grade II	conventional (no special subtype)	Grade II	Grade II (nucleoli visible at x200 magnification)	trabecular 90%, solid 10%	minimal	non	non	very narrow nontumoral liver tissue transition zone detected	non	non	9	non	NA	20	sinusoidal	20	parenchymal	0	NA	20/HPF	15	no specific distribution	non	NA	oui	20	non	non
674 437 T3 PFA 30	noral adjacent	NA	NA	NA	NA	NA	NA	NA	NA	NA	NA	non	non	0	non	NA	6	sinusoidal	3	parenchymal	0	NA	8/HPF	7	no specific distribution	non	NA	non	NA	non	non
673 354 T1 PFA 23	noral adjacent	NA	NA	NA	NA	NA	NA	NA	NA	NA	NA	non	non	0	non	NA	5	sinusoidal	6	parenchymal (3), portal tracts (3)	0	NA	12/HPF	10	no specific distribution	non	NA	non	NA	non	non
673 354 T2 PFA 30	noral adjacent	NA	NA	NA	NA	NA	NA	NA	NA	NA	NA	non	non	0	non	NA	1	sinusoidal	3	parenchymal (1), portal tracts (2)	0	NA	15/HPF	10	no specific distribution	non	NA	non	NA	non	non

NIF: NecroInflammatory Focus HPF: High power field (x40 objective = x400 magnification)

Table S2, related to Fig. 1. Percentage of HCC patients from the indicated cohorts with 55 genes simultaneously overexpressed and hypermethylated in gene body (H+E+ gene) found in *Alb-R26* ^{Met} methylome studies.

gene_name	gene_type	Regulation	TCGA LIHC	LIRI-JP	LICA-FR	HCC NatCom
CDKN2A	HE_genes	up	79,48	77,92	87,5	97,44
GABBR1	HE_genes	up	68,63	12,12	100	4,27
PRRX1	HE_genes	up	65,57	34,63	18,12	87,18
CDKN2B	HE_genes	up	61,79	53,68	81,88	93,16
TMEM191C	HE_genes	up	61,32	24,24	0	13,68
PCDH17	HE_genes	up	60,14	43,29	65	67,52
ARHGAP22	HE_genes	up	59,2	38,96	9,38	60,68
HCN2	HE_genes	up	56,6	38,53	15	47,01
IRX3	HE_genes	up	52,36	51,52	23,12	61,54
DBN1	HE_genes	up	51,18	38,53	17,5	64,1
NFKB2	HE_genes	up	50,94	22,94	1,25	29,06
PTK7	HE_genes	up	48,35	31,6	20,62	48,72
RELB	HE_genes	up	45,28	31,17	10,62	73,5
CLDN7	HE_genes	up	43,16	32,03	8,75	53,85
ADAMTSL5	HE_genes	up	42,92	39,83	1,88	16,24
ANO8	HE_genes	up	42,69	31,17	0	4,27
SSBP4	HE_genes	up	42,69	30,3	0,62	47,01
SRGAP1	HE_genes	up	42,45	32,47	41,88	17,95
SCN8A	HE_genes	up	41,98	31,17	1,88	0
CACNA1B	HE_genes	up	41,27	11,69	0,62	6,84
BHLHA15	HE_genes	up	38,44	26,84	0,62	70,09
DUSP8	HE_genes	up	37,03	29	100	81,2
NEURL1B	HE_genes	up	35,38	25,97	7,5	11,11
HMGN2	HE_genes	up	33,73	30,74	2,5	7,69
FOXC1	HE_genes	up	33,02	18,61	33,75	83,76
EFNB2	HE_genes	up	31,84	27,27	6,25	23,08
ARHGAP27	HE_genes	up	31,84	17,75	100	2,56
MGAT4A	HE_genes	up	31,37	46,75	28,75	54,7
GATA2	HE_genes	up	30,42	17,32	1,25	37,61
BTBD17	HE_genes	up	29,72	3,03	0,62	11,11
GRB10	HE_genes	up	29,72	22,08	0,62	0,85
POU3F1	HE_genes	up	27,59	16,02	18,75	7,69
MAP3K6	HE_genes	up	27,36	10,82	1,25	0,85
MET	HE_genes	up	26,18	29	49,38	5,13
DYSF	HE_genes	up	25,94	8,23	0	0
LTBP3	HE_genes	up	25	14,72	0,62	10,26
ACTN1	HE_genes	up	24,29	9,52	0,62	8,55
TP53INP1	HE_genes	up	23,82	22,94	9,38	19,66
CDKN1C	HE_genes	up	20,52	6,06	100	36,75
CPEB1	HE_genes	up	19,81	12,55	45	11,11
NEURL1	HE_genes	up	18,63		2,5	5,13
SNX20	HE_genes	up	18,16	13,42	18,75	11,97
CHRNA4	HE_genes	up	17,22	7,79	0,62	0
INHBB	HE_genes	up	16,27	13,85	23,12	60,68
TBC1D2	HE_genes	up	15,09	16,02	0	0,85
C2CD4C	HE_genes	up	14,15	9,09	0	11,11
PTPRS	HE_genes	up	12,97	9,52	1,25	1,71
PRICKLE2	HE_genes	up	11,56	11,26	12,5	0
AMN	HE_genes	up	8,49	12,55	0	5,13
SLC20A1	HE_genes	up	7,55	3,9	1,25	10,26
RUNDC3B	HE_genes	up	5,9	8,23	18,75	7,69
MT1A	HE_genes	up	4,95	3,46	0	4,27
SRD5A2	HE_genes	up	4,25	0		0
TSPAN9	HE_genes	up	4,01	3,03	0	0
JUN	HE_genes	up	3,07	2,16	0	94,87

Table S3, related to Fig. 1. Mice used for bulk RNA-seq studies, with their corresponding ID and age.

mouse cohorts	mouse ID	Tumor ID	weeks	Sample ID	genotype
Alb-R26 ^{Met} livers	679904	-	58	HL_E20_S1	*Alb-R26* ^{Met}
	680502	-	71	HL_E20_S2	*Alb-R26* ^{Met}
	680505	-	69	HL_E20_S3	*Alb-R26* ^{Met}
	679950	-	55	HL_E19_S4	*Alb-R26* ^{Met}
	680004	-	52	HL_E19_S5	*Alb-R26* ^{Met}
	680009	-	55	HL_E20_S6	*Alb-R26* ^{Met}
Alb-R26 ^{Met} advanced HCC	679906	T2	69	T2_E20_S7	*Alb-R26* ^{Met}
	678837	T3	69	T3_E20_S8	*Alb-R26* ^{Met}
	679920	T8	59	T8_E19_S9	*Alb-R26* ^{Met}
	679909	T6	63	T6_E19_S10	*Alb-R26* ^{Met}
	680002	T2	52	T2_E19_S11	*Alb-R26* ^{Met}
	679916	T3	72	T3_E20_S12	*Alb-R26* ^{Met}
	678632	T4	72	T4_E19_S13	*Alb-R26* ^{Met}
	679959	T3	55	T3_E20_S14	*Alb-R26* ^{Met}
	679664	T1	68	T1_E19_S15	*Alb-R26* ^{Met}
	6784766	T2	67	T2_E19_S16	*Alb-R26* ^{Met}
	679109	T7	49	T7_E19_S17	*Alb-R26* ^{Met}
	679109	T11	49	T11_E20_S18	*Alb-R26* ^{Met}

Table S4, related to Fig. 1. List of HCC markers used for transcriptomic comparative studies between HCC mouse and human patients.

gene	AlbR26	TCGA_LIHC	LIRI	LICA	HCC_NatCom
ALB	15,025	13,987	13,304	11,885	20,260
ACTB	11,093	10,289	9,810	7,529	14,291
FGB	10,462	11,245	9,740	9,481	17,738
FABP1	9,619	8,339	9,313	5,811	13,120
FASN	7,594	6,578	5,496	4,425	12,911
PCK1	7,430	5,970	6,203	4,423	11,928
RARRES2	7,659	9,156	8,978	6,447	12,674
HAMP	7,246	2,305	3,354	0,854	6,898
MET	6,857	4,754	4,129	3,701	12,980
GCK	6,554	0,943	0,817	0,328	3,318
LCAT	6,604	4,266	4,014	2,094	8,176
C8A	6,618	6,539	5,547	5,122	12,663
RAB1A	6,041	6,324	5,664	4,625	11,594
GLUL	6,848	7,650	8,016	6,413	15,740
RAB1B	5,952	6,908	5,577	3,996	10,497
CDKN1A	6,363	5,994	5,313	3,669	10,616
CRP	6,160	7,780	6,592	5,547	12,946
PTEN	5,896	3,394	3,468	2,179	12,540
CDH1	5,565	5,202	4,636	3,304	11,948
CCND1	4,918	5,248	4,610	3,223	11,876
HNF4A	5,263	5,894	5,289	4,359	12,442
PCNA	5,176	6,166	5,278	4,459	10,164
AFP	5,137	3,504	3,228	1,576	7,717
SAA1	5,266	7,956	6,771	5,373	12,023
GPC3	4,077	7,024	6,004	4,981	11,996
LAPTM4B	4,254	5,488	4,921	3,667	11,061
VEGFA	5,108	4,061	5,897	2,948	11,478
ESR1	3,875	0,728	0,944	0,442	8,504
CDH2	4,467	4,723	4,097	3,335	11,904
FGFR4	3,826	5,891	5,231	3,393	10,591
SAE1	3,816	5,079	4,364	3,139	10,552
VIM	4,121	6,362	6,939	4,156	12,574
TRP53	3,868	3,460	3,165	1,964	9,162
LYVE1	3,333	1,256	1,260	0,694	7,716
EPHA1	3,311	3,626	2,795	1,693	9,334
HNF1A	3,328	4,093	3,469	2,101	8,824
KDR	3,310	2,779	2,498	1,795	10,980
OAT	3,819	3,549	3,844	2,286	9,963
SOX9	3,198	3,311	2,987	1,781	8,556
FLT1	3,391	2,412	2,209	1,455	11,203
G0S2	3,845	6,127	5,586	3,789	9,583
IGF2	2,065	5,359	4,345	2,168	1,858
MYCN	2,404	0,910	0,612	0,425	5,322
PPP2R1B	2,465	4,534	4,660	3,257	11,840
PROM1	2,902	0,522	0,798	0,174	5,180
MERTK	2,540	2,615	2,927	2,131	10,176
MYC	2,282	4,352	4,128	2,454	8,849

HMGB3	2,324	4,342	3,222	2,328	10,077
CD34	2,210	3,062	3,373	2,186	8,823
CD24A	1,624	5,192	5,028		11,266
NOTCH4	1,837	2,093	2,308	0,964	8,469
HGF	1,553	0,978	1,267	0,622	8,922
CD44	1,839	2,715	3,177	1,708	10,410
CCL5	1,689	3,892	3,499		7,652
LAMA3	1,407	1,623	2,362	1,127	10,311
GMNN	1,805	4,555	4,606	2,935	9,365
JAG1	1,356	2,669	2,750	1,682	10,495
BIRC5	1,664	3,072	2,385	1,300	5,855
DLL1	1,007	1,586	1,359	0,614	5,833
MKI67	1,816	2,120	1,807	1,176	9,723
CDC20	1,294	3,298	2,670	1,378	7,181
ADM	0,930	3,254	3,490	1,614	8,051
KIT	0,897	0,633	0,564	0,311	6,745
AURKA	1,382	3,293	2,981	1,891	8,727
THY1	0,910	3,950	3,908	1,798	9,386
IGF1R	0,982	0,709	0,617	0,269	7,720
NQO1	2,016	4,197	3,121	2,452	9,478
TBX3	1,455	3,304	3,223	2,504	10,366
EPCAM	0,540	2,320	2,268	0,882	5,462
ANGPT2	0,680	1,400	1,153	0,743	8,360
BASP1	0,883	2,147	2,136	0,939	6,614
NOTCH3	0,616	2,928	2,277	1,391	9,045
CD274	0,751	0,774	1,097	0,433	6,704
MDK	0,385	6,130	6,151	3,464	8,948
ANGPT1	0,464	0,761	1,042	0,486	8,166
KRT7	0,347	2,660	2,496	0,888	6,868
LGR5	0,630	1,257	1,425	1,072	5,782
PAK3	0,186	0,213	0,371	0,124	4,555
PTGDS	0,181	3,223	3,525	1,447	6,716
KRT19	0,358	1,834	1,885	0,413	4,555
FCRLA	0,155	0,436	0,507	0,184	3,824
PCSK1	0,075	0,235	0,125	0,025	6,001
VCAN	0,154	1,634	2,392	0,879	11,181
MYH4	0,065	1,411	1,246	0,583	4,306
DLK1	0,261	1,150	1,527	0,305	2,140
CALCA	0,004	0,684	0,686	0,259	3,156
DKK1	0,000	1,529	1,484	0,741	4,577
IGF2BP3	0,087	1,097	1,558	0,536	7,710
FGF15	0,000	0,968	0,971	0,389	2,781
FGF3	0,000	0,058	0,180	0,012	0,099
FGF4	0,000	0,063	1,175	0,032	0,040
IL6	0,014	0,433	0,890	0,183	2,443
PDCD1	0,014	1,032	0,805	0,363	3,119
REG3A	0,000	2,323	2,891	1,553	5,791
NRCAM	0,140	1,038	0,924	0,778	7,145
GP2	0,007	0,554	0,742	0,156	1,897

Table S5, related to Fig. 1. List of immune checkpoints used for transcriptomic comparative studies between HCC mouse and human patients.

gene	AlbR26	TCGA_LIHC	LIRI	LICA	HCC_NatCom
LGALS9	6,91	3,12	3,89	1,03	7,97
FGL1	6,80	8,46	8,47	6,60	15,25
HMGB1	5,69	4,86	6,03	3,40	12,72
CEACAM1	5,07	4,32	4,25	2,88	10,19
NECTIN2	4,08	6,31		3,66	10,69
CD276	2,36	4,27	3,88	2,16	9,73
TNFRSF14	2,60	4,07	4,97	2,36	8,78
PVR	2,47	4,95	3,65	2,96	11,05
TNFRSF18	1,61	1,30	1,07	0,33	2,60
CD40	1,41	3,41	3,33	1,93	8,11
CD200	1,41	1,42	1,58	0,77	6,50
CD274	0,75	0,77	1,10	0,43	6,70
CD80	0,28	0,36	0,64	0,18	5,88
CD200R1	0,54	0,39	0,69	0,25	6,35
LAG3	0,52	1,39	1,51	0,58	3,99
CD86	0,52	1,57	1,91	0,87	8,02
TNFRSF9	0,28	0,47	0,34	0,26	4,42
CD27	0,14	2,29	1,97	0,61	3,95
HAVCR2	0,33	1,74	2,20	0,87	8,23
TNFSF9	0,10	0,74	0,58	0,22	2,33
CD28	0,17	0,73	0,85	0,43	7,25
ICOS	0,09	0,47	0,71	0,25	5,07
CD96	0,05	0,86	0,98	0,47	7,00
TIGIT	0,28	0,66	1,01	0,32	5,51
CD40LG	0,01	0,70	0,64	0,29	4,22
TNFSF14	0,12	2,43	3,89	1,80	8,04
VTCN1	0,18	0,64	0,70	0,22	4,64
TNFRSF4	0,30	2,01	1,65	0,60	3,81
TNFSF18	0,09	0,30	0,36	0,13	2,63
CD226	0,09	0,25	0,53	0,19	6,64
TNFSF4	0,03	1,19	1,23	0,61	6,49
BTLA	0,18	0,21	0,26	0,14	4,78
PDCD1	0,01	1,03	0,81	0,36	3,12
CD70	0,00	0,35	0,47	0,14	1,61
CD160	0,05	0,39	0,84	0,10	5,39
PDCD1LG2	0,14	0,79	0,84	0,40	6,59

Table S6, related to Fig. 2-8. List of antibodies used for the spectral cytometry analysis.

Antigen	Fluorochrome	Clone	Company	Catalogue ref.
CD11b	BUV395	M1/70	BD Horizon	103255
CD8	BUV563	53-6.7	BD Optibuild	748535
CD88	BUV805	20/70	BD Optibuild	748611
or F4/80	BUV805	T45-2342	BD Optibuild	749282
CD24	VioGreen	REA743	Miltenyi	130-110-694
NK1.1	BV570	PK136	BD Horizon	564143
CD45	BV785	30-F11	Biolegend	103149
MHCII	FITC	M5/114.15.2	Invitrogen	11-5321-82
Ly6C	PE Dazzle594	HK1.4	Biolegend	128044
CD11c	PE-Cyn7	N418	Biolegend	117318
Ly6G	AF700	1A8	Biolegend	127621
CD3	APC Vio770	REA641	Miltenyi	130-119-793
**Panel Immune checkpoints**				
CD274/PDL1	BUV615	10F.9G2	Biolegend	752339
CD80	BUV737	16-10A1	BD Horizon	612773
CD40	BV421		BD Optibuild	562846
CD279/PD1	BV605	29F.1A12	Biolegend	135220
CD276/B7-H3	PercP Cyn5.5	MIH35	Biolegend	135615
CD152/CTLA-4	PE	UC10-4B9	Invitrogen	12-1522-82
CD366/TIM3	PE Fire 810	RMT3-23	Biolegend	119745
B220	APC	RA3-6B2	Biolegend	103211
PTK7	Alexa Fluor 647	1H6	Home made	
**2° Panel Immune checkpoints**				
B220	BV711	RA3-6B2	Biolegend	103255
CD278/ICOS	VioBlue	7E.17G9	Miltenyi	130-102-740
LGAL9	PercP Cyn5.5	RG9-35	Biolegend	136111
GITRL	PE	YGL 386	Biolegend	120305
CD275/ICOSL	APC	REA990	Miltenyi	130-116-447
**FMO-isotype control used**				
Hamster IgG2, κ	BV711	B81-3	BD Horizon	612774
Rat IgG2ak	BV605	RTK2758	Biolegend	400540
Rat IgG2ak	Alexa Fluor 647	MRG2a-83	Biolegend	407512

Table S7, related to Fig. 2-8. Mice used for in vivo longitudinal studies. For each cohort, ID and age of mice at the beginning of experiment, number and size of tumours are detailed.

Cohorts	ID mice	Genotype	Age of the mice at the begining of the experiment [weeks]	Number of tumours	Size of the tumours [mm^3]
Control	679274	*Alb-R26*^{Met}	81	6	4.6 to 81
	670323	*Alb-R26*^{Met}	67	3	1 to 1.4
	679551	*Alb-R26*^{Met}	67	1	7.4
	680501	*Alb-R26*^{Met}	64	3	0.5 to 6.7
	679592	*Alb-R26*^{Met}	64	4	1.9 to 48.1
	679763	*Alb-R26*^{Met}	56	1	200
	**tumour cohort**			**18**	**0.5 to 200**
Decitabine (10 days)	679585	*Alb-R26*^{Met}	64	11	0.9 to 17.3
	680504	*Alb-R26*^{Met}	61	3	5 to 200
	679646	*Alb-R26*^{Met}	61	4	7.5 to 70
	679772	*Alb-R26*^{Met}	58	1	2.4
	679832	*Alb-R26*^{Met}	54	5	1 to 250
	679799	*Alb-R26*^{Met}	54	1	220
	**tumour cohort**			**25**	**0.9 to 250**
MEK$_i$+BCL-XL$_i$ (10 days)	678764	*Alb-R26*^{Met}	105	3	3 to 220
	679549	*Alb-R26*^{Met}	67	1	80.5
	679570	*Alb-R26*^{Met}	66	6	2.9 to 86
	680503	*Alb-R26*^{Met}	63	3	0.9 to 160
	679830	*Alb-R26*^{Met}	52	6	5.1 to 291
	679798	*Alb-R26*^{Met}	54	1	5
	**tumour cohort**			**20**	**0.9 to 291**
Decitabine (20 days)	678681	*Alb-R26*^{Met}	60	3	0.2 to 1.7
	676823	*Alb-R26*^{Met}	59	3	2.1 to 192.3
	676841	*Alb-R26*^{Met}	58	3	0.1 to 0.5
	678686	*Alb-R26*^{Met}	58	3	0.1 to 8.6
	676879	*Alb-R26*^{Met}	57	4	0.4 to 1.3
	**tumour cohort**			**16**	**0.1 to 192.3**
MEK$_i$+BCL-XL$_i$ (20 days)	678683	*Alb-R26*^{Met}	60	3	0.6 to 71.9
	676852	*Alb-R26*^{Met}	58	5	0.2 to 1
	678687	*Alb-R26*^{Met}	48	3	0.8 to 42.5
	677248	*Alb-R26*^{Met}	48	3	0.3 to 1.3
	678688	*Alb-R26*^{Met}	49	2	0.4 to 2
	**tumour cohort**			**16**	**0.2 to 71.9**
Decitabine + ICI (20 days)	675811	*Alb-R26*^{Met}	70	8	0.8 to 101.8
	677411	*Alb-R26*^{Met}	68	4	0.1 to 5.1
	675830	*Alb-R26*^{Met}	69	2	1.3 to 5.6
	677946	*Alb-R26*^{Met}	63	5	0.9 to 10.1
	677947	*Alb-R26*^{Met}	63	2	0.5 to 7.4
	677424	*Alb-R26*^{Met}	65	2	1.3 to 2.5
	677698	*Alb-R26*^{Met}	66	2	1.1 to 5.3
	677703	*Alb-R26*^{Met}	62	4	1.5 to 16.7
	676069	*Alb-R26*^{Met}	62	2	1.6 to 4.1
	**tumour cohort**			**31**	**0.1 to 101.8**
MEK$_i$+BCL-XL$_i$ + ICI (20 days)	677402	Alb-R26^{Met}	73	2	2.9 to 7.5
	675793	Alb-R26^{Met}	71	3	4.5 to 239.2
	677412	Alb-R26^{Met}	68	3	0.8 to 29.2
	675948	Alb-R26^{Met}	65	5	0.7 to 28.2
	675961	Alb-R26^{Met}	65	1	2.3
	677699	Alb-R26^{Met}	66	5	1.2 to 115
	677700	Alb-R26^{Met}	65	6	1 to 76.9
	677704	Alb-R26^{Met}	62	2	1.7 to 4.6
	676070	Alb-R26^{Met}	62	1	4.3
	**tumour cohort**			**28**	**0.7 to 239.2**

Table S8, related to Fig. 2-8. Mice used for longitudinal studies with tumour localization and state. ML: Median Lobe; LLL: Left Lateral Lobe; RLL: right lateral Lobe; CL: Caudal lobe.

Cohorts	ID mice	Mouse age at the begining of the experiment [weeks]	ID tumour	State	Localisation
Control	679274	81	T1	Evolutive	ML
			T2	Stable	ML
			T3	Evolutive	ML
			T4	Evolutive	ML
			T5	Evolutive	LLL
			T6	Evolutive	LLL
	670323	67	T1	Evolutive	ML
			T2	Evolutive	RLL
			T3	Evolutive	LLL
	679551	67	T1	Evolutive	ML
	680501	64	T1	Regressive	ML
			T2	Stable	ML
			T3	Evolutive	ML
	679592	64	T1	Evolutive	ML
			T2	Evolutive	LLL
			T3	Stable	RLL
			T4	Stable	RLL
	679763	56	T1	Regressive	LLL
Decitabine 10 days	679585	64	T1	Regressive	ML
			T2	Evolutive	ML
			T3	Regressive	ML
			T4	Evolutive	LLL
			T5	Evolutive	ML
			T6	Evolutive	ML
			T7	Stable	LLL
			T8	Evolutive	ML
			T9	Regressive	ML
			T10	Evolutive	ML
			T11	Stable	LLL
	680504	61	T1	Evolutive	ML
			T2	Evolutive	ML
			T3	Evolutive	LLL
	679646	61	T1	Evolutive	ML
			T2	Stable	ML
			T3	Evolutive	ML
			T4	Regressive	RLL
	679772	58	T1	Stable	ML
	679832	54	T1	Stable	ML
			T2	Evolutive	ML
			T3	Stable	LLL
			T4	Regressive	LLL
			T5	Regressive	ML
	679799	54	T1	Evolutive	ML
	678764	105	T1	Regressive	ML
			T2	Regressive	ML
			T3	Regressive	RLL
	679549	67	T1	Regressive	LLL
	679570	66	T1	Regressive	ML
			T2	Regressive	ML
			T3	Regressive	ML

MEKi+BCL-XLi 10 days	[illegible]	[illegible]	T4	Evolutive	LLL
			T5	Stable	ML
			T6	Regressive	RLL
	680503	63	T1	Regressive	ML
			T2	Regressive	ML
			T3	Stable	ML
	679830	52	T1	Regressive	ML
			T2	Regressive	ML
			T3	Regressive	LLL
			T4	Regressive	ML
			T5	Regressive	ML
			T6	Regressive	RLL
	679798	54	T1	Regressive	RLL
Decitabine 20 days	678681	60	T2	Evolutive	ML
			T3	Evolutive	ML
			T4	Stable	RLL
	676823	59	T1	Evolutive	ML
			T2	Evolutive	LLL
			T3	Evolutive	ML
	676841	58	T1	Evolutive	ML
			T2	Regressive	LLL
			T3	Regressive	LLL
	678686	58	T0	Stable	ML
			T2	Evolutive	LLL
			T3	Stable	LLL
	676879	57	T1	Stable	ML
			T2	Regressive	ML
			T3	Evolutive	ML
			T4	Evolutive	LLL
MEKi+BCL-XLi 20 days	678683	60	T1	Regressive	ML
			T2	Regressive	ML
			T3	Regressive	CL
	676852	58	T1	Regressive	ML
			T2	Regressive	LLL
			T3	Regressive	ML
			T4	Regressive	ML
			T5	Regressive	ML
	678687	48	T1	Regressive	ML
			T2	Regressive	ML
			T3	Regressive	ML
	677248	48	T1	Regressive	ML
			T2	Regressive	LLL
			T3	Regressive	CL
	678688	49	T1	Regressive	ML
			T2	Regressive	RLL
	675811	70	T1	Stable	ML
			T2	Evolutive	LG
			T5	Evolutive	LG
			T6	Evolutive	ML
			T7	Evolutive	RLL
			T8	Regressive	RLL
			T9	Regressive	RLL
	677411	68	T1	Stable	ML
			T2	Regressive	RLL

Decitabine + immuno			T3	Evolutive	ML
	675830	69	T1	Regressive	ML
			T2	Regressive	LLL
	677946	63	T1	Regressive	ML
			T2	Evolutive	ML
			T3	Evolutive	ML
			T4	Evolutive	RLL
			T5	Regressive	LLL
	677947	63	T1	Regressive	ML
			T2	Regressive	CL
	677424	65	T1	Evolutive	ML
			T2	Evolutive	RLL
	677698	66	T1	Stable	ML
			T2	Regressive	ML
			T3	Regressive	CL
	677703	62	T1	Stable	ML
			T2	Regressive	ML
			T4	Regressive	LLL
			T5	Evolutive	RLL
	676069	62	T1	Regressive	ML
			T2	Regressive	LLL
MEK+BCL-XL + immuno	677402	73	T1	Regressive	ML
			T2	Regressive	LLL
	675793	71	T1	Regressive	ML
			T2	Regressive	ML
			T3	Regressive	RLL
	677412	68	T1	Regressive	ML
			T2	Regressive	ML
			T3	Regressive	LLL
	675948	65	T1	Stable	ML
			T2	Regressive	LLL
			T3	Evolutive	CL
			T6	Regressive	ML
			T7	Regressive	ML
	675961	65	T1	Regressive	ML
	677699	66	T1	Regressive	ML
			T2	Regressive	LLL
			T3	Regressive	ML
			T4	Regressive	LLL
			T5	Regressive	CL
	677700	65	T1	Evolutive	ML
			T2	Regressive	ML
			T3	Regressive	LLL
			T4	Regressive	LLL
			T5	Stable	ML
			T6	Regressive	LLL
	677704	62	T1	Regressive	ML
			T2	Regressive	LLL
	676070	62	T1	Regressive	ML

SUPPLEMENTARY TABLES

Table S1, related to Fig. 1 and S1. Pathological analysis of HES tumours and liver samples from *Alb-R26*^{Met} mice.

Table S2, related to Fig. 1. Percentage of HCC patients from the indicated cohorts with alterations of genes found in *Alb-R26*^{Met} transposon studies[27]: overexpression of 29 predicted oncogenes, downregulation of 84 predicted tumour suppressors, and altered expression of 137 deregulated genes.

Table S3, related to Fig. 1. Mice used for bulk RNA-seq studies, with their corresponding ID and age.

Table S4, related to Fig. 1. List of HCC markers used for transcriptomic comparative studies between HCC mouse and human patients.

Table S5, related to Fig. 1. List of immune checkpoints used for transcriptomic comparative studies between HCC mouse and human patients.

Table S6, related to Fig. 2-8. List of antibodies used for the spectral cytometry analysis.

Table S7, related to Fig. 2-8. Mice used for in vivo longitudinal studies. For each cohorts, ID and age of mice at the beginning of the experiment, number and size of tumours are detailed.

Table S8, related to Fig. 2-8. Mice used for longitudinal studies with tumour localization and state. For each cohort, ID, state (evolutive or regressive), and localization of tumours at the end of the experiment are detailed.
